# Targeting osteoblast fatty acid metabolism attenuates skeletal and cardiovascular complications in chronic kidney disease models

**DOI:** 10.1172/JCI207322

**Published:** 2026-09-29

**Authors:** Petra Simic, Han Xie, Wen Zhou, Yu Fan, Renata C. Pereira, Fangcong Dong, Jason D. Roh, Isidro B. Salusky, Charandeep Singh, Russell P. Goodman, Ashok Khatri, Eugene P. Rhee

**Affiliations:** 1 Nephrology Division, Mass General Brigham, Boston, Massachusetts, USA.; 2 Endocrine Unit, Massachusetts General Hospital, Boston, Massachusetts, USA.; 3 Department of Pediatrics, Division of Pediatric Nephrology, UCLA David Geffen School of Medicine, Los Angeles, California, USA.; 4 Department of Molecular Biology and; 5 Cardiovascular Research Center, Massachusetts General Hospital, Boston, Massachusetts, USA.; 6 Department of Ophthalmology, Tufts Medical Center, Tufts University School of Medicine, Boston, Massachusetts, USA.; 7 Liver Center, Division of Gastroenterology, Massachusetts General Hospital, Boston, Massachusetts, USA.

**Keywords:** Bone biology, Cardiology, Nephrology, Bone disease, Cardiovascular disease, Chronic kidney disease

## Abstract

Chronic kidney disease (CKD) disrupts mineral homeostasis, leading to impaired skeletal mineralization and cardiovascular pathology, yet the mechanism linking these processes remains undefined. Here, we identify glycerol-3-phosphate acyltransferase 2 (GPAT2) as a regulator that couples free fatty acid (FFA) partitioning in osteoblasts to systemic phosphate balance. In mouse and human CKD, elevated osteoblast GPAT2 routed FFA away from mitochondrial oxidation, limiting phosphate incorporation into bone and toward lysophosphatidic acid synthesis, increasing production of the phosphaturic hormone FGF23. By contrast, osteoblast-specific *Gpat2* deletion restored osteoblast FFA oxidation and skeletal phosphate incorporation, lowered circulating phosphate and FGF23, and attenuated vascular calcification and cardiac hypertrophy in CKD. Furthermore, a bone-targeted GPAT inhibitor recapitulated most of these beneficial effects. These findings establish a fundamental role for osteoblast lipid metabolism in mineral homeostasis and identify GPAT2 in bone as a promising therapeutic target for both skeletal and cardiovascular complications of CKD.

## Abstract 2

This research identifies how kidney disease harms bones and the cardiovascular system and reveals a potential treatment.

## Graphical abstract

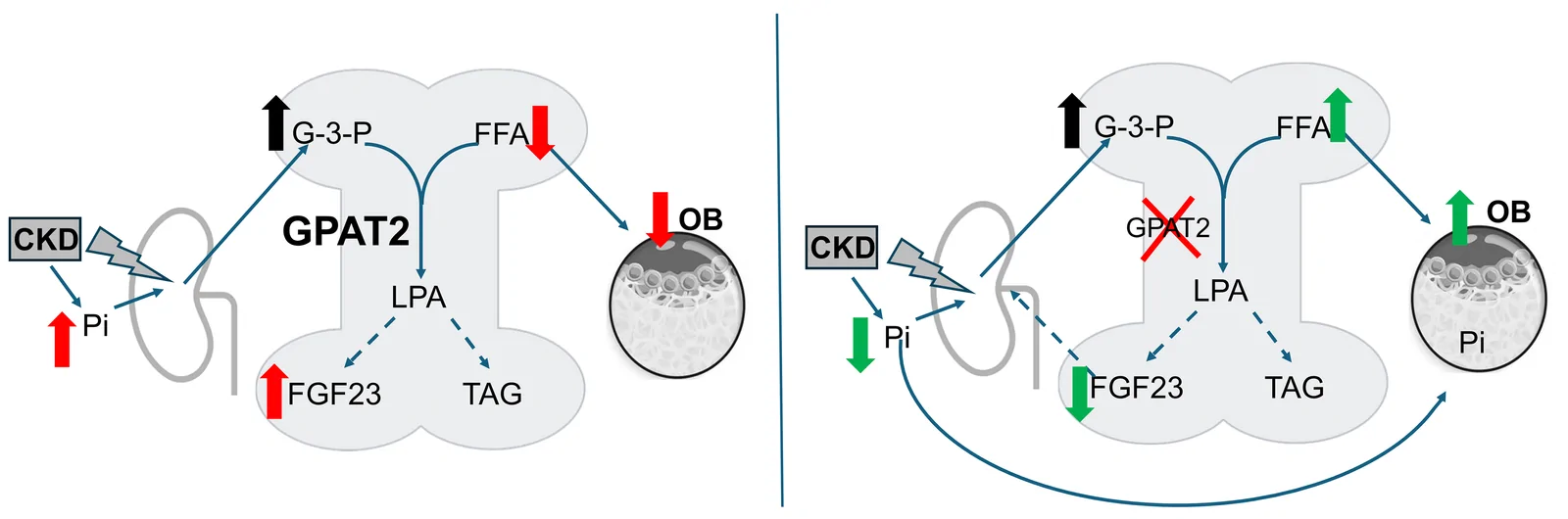

## Introduction

Mineral homeostasis is essential for life and depends on a coordinated process that integrates nutrient availability and endocrine signaling across multiple organs. Bone functions as the dominant site of phosphate flux, actively coupling osteoblast metabolic activity to mineral incorporation rather than serving as a passive structural reservoir. In chronic kidney disease (CKD), this metabolic integration collapses, such that impaired skeletal mineralization occurs in parallel with progressive vascular calcification and cardiac hypertrophy, a constellation termed CKD–mineral and bone disorder (CKD-MBD) (1, 2). The basis of this mineralization paradox remains poorly defined (3, 4).

In CKD, skeletal abnormalities emerge early, preceding detectable changes in circulating calcium, phosphate, FGF23, parathyroid hormone (PTH), or vitamin D (5, 6). As kidney function declines, the skeleton progressively loses its capacity to incorporate phosphate into newly formed bone matrix, reducing skeletal buffering and allowing phosphate to accumulate in the circulation (7, 8). Elevated phosphate drives vascular calcification, while increased FGF23 — initially adaptive by promoting urinary phosphate excretion — may become maladaptive, as it has been associated with cardiac hypertrophy and may contribute to its development (9–12). Current therapies focus on correcting circulating mineral levels or targeting individual hormonal pathways, yet they fail to reduce fracture risk or cardiovascular mortality and, in some cases, exacerbate mineral imbalance; for example, experimental FGF23 neutralization was expected to mitigate cardiac hypertrophy but worsened phosphate retention and vascular calcification, leading to increased mortality (6, 13–16). Furthermore, therapy with active vitamin D sterols is associated with persistent bone mineralization defects (17, 18). These limitations highlight an important gap, reflecting the absence of strategies that restore the capacity of bone to utilize phosphate.

Osteoblasts rely heavily on free fatty acid (FFA) metabolism for energy, with fatty acid oxidation (FAO) providing 40%–80% of osteoblast ATP (19–22). The skeleton is a major consumer of circulating lipids, second only to the liver, and bone uptake of lipid particles rivals that of hepatocytes (22, 23). Disrupting FAO in osteoblasts impairs differentiation and mineralization, whereas enhancing FFA availability promotes osteoblastogenesis and bone formation in vivo (20, 22, 24). CKD is characterized by systemic alterations in lipid metabolism, including reduced FAO and elevated circulating triglycerides (25, 26). However, how FFA utilization is regulated within osteoblasts — and whether dysregulated FFA routing directly limits mineralization in CKD — has not been examined.

Glycerol-3-phosphate acyltransferase 2 (GPAT2), the predominant GPAT isoform expressed in bone, catalyzes the rate-limiting step in triglyceride synthesis, incorporating a FFA into glycerol-3-phosphate to produce lysophosphatidic acid (LPA) (27, 28). Recently, we identified a kidney-bone signaling axis in which kidney glycerol-3-phosphate (G-3-P), released in response to phosphate loading or kidney injury, circulates to bone and stimulates GPAT2-dependent LPA production, which then increases FGF23 expression (28, 29). Here, we test the hypothesis that osteoblast GPAT2 functions as a metabolic gatekeeper that controls bone FFA partitioning in CKD. We show that CKD drives GPAT2-dependent diversion of FFA away from osteoblast mitochondrial oxidation toward LPA synthesis, limiting phosphate incorporation into bone and inducing FGF23 expression and its downstream cardiovascular pathology. Conversely, restricting GPAT2 activity restores osteoblast FAO, enhances skeletal phosphate incorporation, and protects against vascular calcification and cardiac hypertrophy. Together, these findings identify GPAT2-dependent metabolic reprogramming in bone as a therapeutic approach that may simultaneously ameliorate all aspects of CKD-MBD.

## Results

### Osteoblast Gpat2 regulates FGF23 production and blood phosphate levels.

GPAT2 mediates the effect of G-3-P, a kidney-derived signaling metabolite, on FGF23 production in primary osteoblasts derived from bone marrow mesenchymal progenitors in vitro (Figure 1A) (28). To investigate the in vivo role of GPAT2 in mineral metabolism, we generated *Gpat2*^fl/fl^ mice (Figure 1B) and bred them with *Osx*-cre mice to achieve osteoblast lineage–specific deletion of *Gpat2* (O-*Gpat2*^–/–^). Recombination occurred in both cortical and trabecular bone (Figure 1C), resulting in approximately 90% reduction of *Gpat2* expression (Supplemental Figure 1, A and B; supplemental material available online with this article; https://doi.org/10.1172/JCI207322DS1). This was accompanied by partial compensatory upregulation of *Gpat1* and *Gpat3* in bone marrow adipocytes in O-*Gpat2*^–/–^ mice. In contrast, bone tissue and osteoblasts showed no such compensation and instead trended toward reduced expression of other *Gpats*, a finding unlikely to be biologically meaningful given their low baseline expression in bone (Supplemental Figure 1, C and D).

At baseline, O-*Gpat2*^–/–^ mice exhibited elevated circulating G-3-P, reduced FGF23 levels, and decreased bone *Fgf23* expression (Figure 1, D and E, and Supplemental Figure 2A). Despite this, these mice had a modest but significant reduction in plasma phosphate and unchanged fractional phosphate excretion (Figure 1, F and G). Plasma PTH, 1,25-dihydroxyvitamin D_3_, and calcium levels remained unchanged (Supplemental Figure 2B). To affirm the involvement of GPAT2 in the G-3-P/LPA/FGF23 pathway (Figure 1A) (28, 29) we i.p. injected O-*Gpat2*^–/–^ mice and their littermate controls with G-3-P or LPA, the substrate and product of the GPAT2-catalyzed reaction, respectively. G-3-P increased FGF23 levels in WT mice but not in O-*Gpat2*^–/–^ mice (Figure 1H), whereas LPA administration increased FGF23 in both genotypes (Figure 1I), confirming that GPAT2 is required for G-3-P–driven LPA production and FGF23 induction in vivo. O-*Gpat2*^–/–^ mice exhibited normal bone structure, with comparable bone volume, trabecular and cortical thickness, and osteoblast, osteocyte, and osteoclast numbers to their O-*Gpat2*^+/+^ littermates (Supplemental Figure 2, C and D).

Taken together, these findings establish osteoblast *Gpat2* as a regulator of FGF23 production and systemic phosphate balance.

### GPAT2 limits osteoblast FFA availability, oxidative metabolism, and mineralization.

Because GPAT2 consumes FFAs during LPA synthesis, we next examined whether *Gpat2* deletion alters intracellular lipid partitioning in osteoblasts. Primary osteoblasts isolated from O-*Gpat2*^–/–^ mice showed increased FFA levels, reduced LPA levels, and reduced *Fgf23* expression (Figure 2, A–C). Conversely, treatment of WT osteoblasts with G-3-P increased *Gpat2* expression, decreased FFA levels, and increased LPA and FGF23 production (Supplemental Figure 3, A–D).

Furthermore, increased FFA availability in O-*Gpat2*^–/–^ osteoblasts was associated with increased mitochondrial oxidative metabolism, as demonstrated by an increase in maximal oxygen consumption rate (OCR) compared with WT cells (Figure 2D). Consistent with the established requirement for FFA in osteoblast differentiation (19), O-*Gpat2*^–/–^ osteoblasts formed more mineralized nodules, exhibited increased expression of mineralization-associated genes, and, importantly, consumed more phosphate from the culture medium (Figure 2, E–H, and Supplemental Figure 4, A and B). Pharmacologic inhibition of FAO with etomoxir abolished mineralization in both WT and O-*Gpat2*^–/–^ osteoblasts (Figure 2, I and J).

Together, these data indicate that GPAT2 limits osteoblast FFA oxidation and mineralization (i.e., phosphate utilization) under physiological conditions, raising the possibility that pathological upregulation of GPAT2 may contribute to impaired bone mineralization in disease states such as CKD.

### CKD increases GPAT2 expression and activity in human and mouse osteoblasts.

CKD is associated with elevated circulating G-3-P levels in both humans and mice (30–32), prompting us to test whether this metabolic milieu alters osteoblast GPAT2 expression and activity. Primary human osteoblasts derived from bone biopsies of 6 patients with advanced CKD exhibited a 4-fold increase in *GPAT2* expression compared with bone cells from healthy controls (*n* = 3) (Figure 3A). Consistent with increased GPAT2 activity, osteoblasts from these patients with CKD exhibited reduced intracellular FFA levels, as reflected by a decreased abundance of the predominant species palmitate and stearate, accompanied by a corresponding increase in LPA production (Figure 3, B and C).

Similarly, primary osteoblasts isolated from mice subjected to adenine diet–induced CKD for 4 weeks showed increased *Gpat2* expression, reduced intracellular FFAs, elevated LPA levels, and reduced cellular respiration compared with controls (Figure 3, D–G).

These findings identify CKD as a state of increased osteoblast GPAT2 activity and suggest that enhanced diversion of FFAs toward LPA synthesis may contribute to defective mineralization and disordered phosphate handling in CKD.

### Osteoblast-specific Gpat2 deletion preserves mineral and bone metabolism in CKD.

To determine whether limiting osteoblast GPAT2 activity mitigates CKD-associated mineral abnormalities in vivo, we subjected O*-Gpat2*^–/–^ mice and littermate controls to adenine diet–induced CKD. O*-Gpat2*^–/–^ mice developed kidney injury to a similar extent as controls, as indicated by comparable increases in plasma creatinine during CKD progression (Figure 4A). G-3-P levels increased with CKD in both genotypes; although O*-Gpat2*^–/–^ mice exhibited elevated baseline G-3-P, levels rose to those observed in WT mice following adenine feeding (Figure 4B).

Consistent with reduced LPA synthesis in the absence of osteoblast *Gpat2*, circulating FGF23 levels and bone *Fgf23* expression were lower in O*-Gpat2*^–/–^ mice with CKD (Figure 4C and Supplemental Figure 5A). Despite reduction of this phosphaturic hormone and reduced urinary fractional phosphate excretion, O*-Gpat2*^–/–^ mice exhibited significantly lower serum phosphate concentrations (Figure 4, D and E), indicating a dissociation between FGF23 signaling and systemic phosphate levels. Other systemic parameters of mineral metabolism did not differ between genotypes at the study endpoint (Supplemental Figure 5B), and circulating triglyceride levels were similar at baseline and during CKD (Supplemental Figure 6).

Reduced serum phosphate concentrations despite lower FGF23 levels and reduced fractional phosphate excretion raised the possibility of enhanced skeletal phosphate incorporation in O*-Gpat2*^–/–^ mice. Consistent with this, histomorphometric and dynamic bone analyses showed that O*-Gpat2*^–/–^ mice with adenine-induced CKD exhibited increased bone volume and osteoblast number, along with enhanced mineralization, higher bone formation and mineral apposition rates, and reduced osteoid thickness compared with controls (Figure 4, F–I, and Supplemental Table 1).

Thus, osteoblast-specific *Gpat2* deletion uncouples reductions in FGF23 from elevated circulating phosphate levels by preserving phosphate utilization for skeletal mineralization in CKD.

### Osteoblast Gpat2 deletion protects against CKD-associated cardiac and vascular disease.

Because elevated FGF23 is associated with cardiac hypertrophy in CKD, therapeutic suppression of FGF23 signaling has been proposed as a strategy to improve cardiovascular outcomes. However, such approaches typically exacerbate hyperphosphatemia and accelerate vascular calcification by impairing renal phosphate excretion (2, 10, 16, 33–35). Having established that O*-Gpat2^–/–^* lowers FGF23 while simultaneously enhancing skeletal phosphate buffering, we next asked whether this metabolic reprogramming confers protection against CKD-associated cardiac and vascular disease.

At baseline, O*-Gpat2^–/–^* mice showed no difference in left ventricular wall thickness compared with littermate controls (Supplemental Table 2), consistent with similarly low circulating FGF23 levels under physiological conditions. In adenine diet–induced CKD, however, O*-Gpat2^–/–^* mice showed significantly reduced interventricular septal diameter, left ventricular wall thickness, and expression of cardiac hypertrophy–associated genes (*Myh7* and *Nppa*) compared with O*-Gpat2^+/+^* mice (Figure 5, A–E, and Supplemental Table 2), in parallel with lower circulating FGF23 levels.

To evaluate vascular outcomes under conditions that promote calcification, we extended the adenine diet and supplemented dietary phosphate to exacerbate mineral stress. Under these conditions, O*-Gpat2^–/–^* mice exhibited significantly reduced vascular mineralization and lower expression of osteogenic marker genes in the vasculature compared with controls (Figure 5, F and G).

To determine whether these effects are independent of CKD model, we confirmed the key findings in a genetic model of CKD. In *Col4a3*^–/–^ (Alport) mice, osteoblast-specific *Gpat2* deletion did not alter the severity of kidney injury but was associated with lower circulating FGF23 and phosphate levels (Supplemental Figure 7, A–G), enhanced osteoblast mineralization (Supplemental Figure 8, A–C), and reduced expression of vascular calcification–related genes (Supplemental Figure 8D). No cardiac hypertrophy was detected in either genotype at this early disease stage, consistent with the young age at analysis (Supplemental Figure 8, E–G).

Together, these data indicate that limiting osteoblast GPAT2 activity mitigates cardiac hypertrophy and vascular calcification, 2 cardinal features of CKD-MBD, thus linking improved skeletal phosphate utilization to protection from downstream cardiovascular disease.

### Gpat2 deficiency increases mineralization by increasing osteoblast FAO.

Because osteoblast-specific *Gpat2* deletion increases skeletal phosphate utilization and protects against cardiovascular complications of CKD, we next defined the underlying metabolic mechanism. In CKD, increased GPAT2 activity in osteoblasts diverted FFAs toward glycerolipid synthesis, resulting in intracellular FFA depletion and accumulation of LPA (Figure 3). In contrast, osteoblasts isolated from O*-Gpat2^–/–^* mice with CKD exhibited restored intracellular FFA levels and reduced LPA abundance and *Fgf23* expression (Figure 6, A–D). Consistent with this shift in FFA availability, mitochondrial oxidative metabolism was significantly increased in O*-Gpat2^–/–^* versus O*-Gpat2^+/+^* osteoblasts from CKD animals (Figure 6E).

To directly test whether FFA availability and FAO limit osteoblast function in CKD, we manipulated lipid metabolism in vitro. Culture in lipid-reduced media markedly suppressed mineralization in both control and CKD osteoblasts, an effect rescued by supplementation with a FFA mixture conjugated to BSA (Figure 6F). Enhancing FFA uptake through overexpression of *Ffar4* similarly increased mineralization in CKD osteoblasts (Figure 6G and Supplemental Figure 9). By contrast, knockdown of *Cpt2*, a key FAO enzyme, abolished the mineralization advantage conferred by *Gpat2* deletion, indicating that FAO acts downstream of GPAT2 (Figure 6G and Supplemental Figure 9). Pharmacologic perturbation yielded concordant results: the FFA receptor agonist GW9508 enhanced mineralization, whereas the FAO inhibitor etomoxir suppressed it in CKD osteoblasts (Figure 6H). Notably, genetic or pharmacologic manipulation of FFA uptake or FAO eliminated differences between *Gpat2*-deficient and control osteoblasts, indicating that altered FFA availability and oxidation are sufficient to override the effects of *Gpat2* loss (Figure 6, F–H).

We next assessed whether local FFA supply is sufficient to restore osteogenic signaling in vivo, comparing the effect of intrafemoral administration of a FFA mixture with vehicle delivery to the contralateral femur. Within 24 hours, expression of *Runx2* and *Alp* increased in CKD bone in response to FFA, exceeding levels observed in FFA-treated control mice (Figure 6, I and J).

Collectively, these data identify GPAT2 as a metabolic gatekeeper that limits osteoblast FAO in CKD. By diverting FFAs away from oxidation toward glycerolipid synthesis, GPAT2 reduces phosphate incorporation into bone, whereas its loss restores FAO-dependent mineralization capacity.

### Bone-targeted GPAT inhibition preserves mineral homeostasis in CKD.

Because GPAT2-mediated diversion of FFA away from oxidation limits osteoblast mineralization in CKD, we next evaluated whether this metabolic constraint can be therapeutically reversed. The small-molecule GPAT inhibitor FSG67 inhibits all GPAT isoforms (GPAT1–4) and enhances FAO, but its systemic activity imposes a narrow therapeutic window in kidney injury, likely due to off-target inhibition of hepatic GPAT1 (28, 36, 37). We therefore developed a bone-targeted GPAT inhibitor to selectively alter osteoblast fatty acid metabolism while minimizing systemic exposure. A bone-targeted GPAT inhibitor was generated by conjugating FSG67 to poly–aspartic acid, which binds hydroxyapatite (43), and tetramethylrhodamine for tracking, yielding bone-GPATi (Figure 7A). Following i.p. administration, bone-GPATi selectively localized to cortical and trabecular bone and colocalized with GPAT2, without detectable hepatic accumulation (Figure 7, B–D). Bone-GPATi blocked G-3-P–stimulated FGF23 production in vivo (Figure 7E).

In adenine diet–induced CKD, bone-GPATi was well tolerated across a wide dose range, with a trend toward reduced FGF23 levels at doses of 1–10 mg/kg, measured 24 hours after a single i.p. injection (Figure 7F), and no evidence of acute hepatic toxicity (Figure 7G). Daily therapeutic dosing lowered circulating FGF23 and phosphate, and reduced fractional phosphate excretion, without altering PTH, calcium, or 1,25-dihydroxyvitamin D_3_ levels throughout adenine-induced CKD (Figure 7, H–J, and Supplemental Figure 10, A–C). Consistent with restored osteoblast FFA availability and metabolism, bone-GPATi enhanced osteoblast mineralization ex vivo (Figure 7, K and L), showed a trend toward increased bone volume in vivo (Supplemental Figure 10, D and E), and markedly attenuated vascular calcification, as indicated by reduced aortic mineral content and osteogenic gene expression (Figure 7, M and N). Cardiac hypertrophy was not observed in this short-duration CKD model (Supplemental Figure 11).

Together, these findings demonstrate that bone-targeted GPAT inhibition therapeutically preserves osteoblast phosphate utilization and mineral homeostasis in CKD, translating osteoblast metabolic reprogramming into systemic protection from pathological mineral redistribution.

## Discussion

CKD disrupts mineral homeostasis, with a reduction in skeletal mineral accumulation coupled to an increase in vascular mineralization. In this study, we identify *Gpat2* as a key metabolic checkpoint in osteoblasts that underlies this paradox. More specifically, increased osteoblast GPAT2 activity in CKD diverts FFA from mitochondrial oxidation toward glycerolipid synthesis, thereby constraining skeletal phosphate incorporation, increasing extraskeletal phosphate levels, and amplifying FGF23-driven cardiovascular pathology. These findings advance our understanding of mineral homeostasis, in health and disease, and position osteoblast lipid metabolism as a disease-modifying node in CKD-MBD.

Recently, we identified kidney glycolysis as the mammalian phosphate sensor upstream of bone FGF23 production (27, 28). In this mechanism, phosphate is first reabsorbed across the apical surface of kidney proximal tubule cells, where it then enters glycolysis as a substrate in the reaction catalyzed by GAPDH. An increase in phosphate, as occurs with dietary phosphate loading, increases glycolytic flux and synthesis of the glycolytic by-product G-3-P. In turn, G-3-P circulates to bone, where GPAT2 combines G-3-P with a FFA to yield LPA, which subsequently stimulates bone FGF23 expression. Because FGF23 acts in the kidney to reduce apical phosphate reabsorption in the proximal tubule, this mechanism constitutes a classic endocrine feedback loop. Consistent with this model, the reduction in FGF23 that results from osteoblast-specific loss of *Gpat2* results in increased G-3-P levels, likely because increased kidney phosphate reabsorption stimulates G-3-P production (it is also possible that G-3-P accumulates because of reduced metabolism by GPAT2).

Whereas our prior work focused on the role of kidney-derived G-3-P in systemic mineral homeostasis, the current study defines a fundamental role for osteoblast FFA partitioning. As noted, when G-3-P and FFA combine to form LPA, there is an increased production of the phosphaturic hormone FGF23 and, thus, net phosphate excretion from the body. By contrast, *Gpat2* inactivation increases FFA availability for mitochondrial oxidation, resulting in increased osteoblast-driven bone mineralization and, thus, net phosphate retention. In support of this, we find that primary osteoblasts from O*-Gpat2^–/–^* mice have higher FFA levels, mitochondrial respiration, and phosphate consumption than their O*-Gpat2^+/+^* counterparts. In turn, O*-Gpat2^–/–^* mice have lower blood phosphate levels, despite lower FGF23 and fractional phosphate excretion. Although we did not observe an increase in bone volume in O*-Gpat2^–/–^* mice under basal conditions, the sensitivity to detect differences may be limited in this context.

CKD is characterized by impaired FAO in the kidney (38, 39). Extending this paradigm, we show that osteoblast FAO is also suppressed in CKD, with G-3-P serving as the key link between altered metabolism in kidney and bone. We have previously shown that increased kidney glycolysis in response to phosphate, as well as AKI and CKD, increases G-3-P production (28, 29). Herein, we show that G-3-P acts as a metabolic signal that channels osteoblast FFAs toward glycerolipid synthesis via GPAT2, at the expense of osteoblast mitochondrial oxidation. Notably, in addition to serving as a substrate for GPAT2, we find that G-3-P increases *Gpat2* expression, such that chronic G-3-P elevations, as occur with CKD, may cause sustained shifts in FFA metabolism, i.e., away from osteoblast FAO. More work is required to understand how G-3-P regulates the expression of *Gpat2*. In addition, we note that phosphate is known to have direct effects on bone mineralization; whether this is through a G-3-P–independent effect on *Gpat2* warrants further investigation.

An important unresolved question is whether the FFAs utilized by GPAT2 derive predominantly from systemic circulation or the bone marrow niche and, in turn, whether modulating extracellular FFAs can influence GPAT2-driven mineral dysregulation in CKD. Prior studies manipulating systemic FFA availability — through dietary enrichment or in vitro FFA supplementation — have yielded heterogeneous effects on bone metabolism. Although higher total FFA intake has been associated with increased bone mineral density in some adult cohorts, analyses of individual fatty acid species reveal divergent relationships with osteoporosis risk, and in vitro studies demonstrate dose-dependent effects on osteoblast differentiation, with low concentrations supporting osteogenesis and higher levels inducing inhibitory or proinflammatory responses (40–47). These inconsistencies likely reflect the metabolic context in which FFAs are processed rather than absolute lipid supply. Importantly, systemic FFA elevation perturbs whole-body metabolism and is linked to cardiovascular disease, limiting both the interpretability and translational potential of global lipid supplementation strategies (48).

How the bone marrow microenvironment regulates GPAT2 activity warrants further investigation. Bone marrow adipose tissue is increasingly recognized as a metabolically active compartment that modulates skeletal homeostasis (49, 50). Increased marrow adiposity accompanies reduced bone mineralization and is a hallmark of aging, osteoporosis, and metabolic disorders, including type 2 diabetes and CKD (51–53). In response to systemic cues and nutrient stress, mesenchymal progenitors preferentially undergo adipogenic differentiation at the expense of osteogenesis, promoting bone marrow adipocyte expansion and impairing skeletal mineralization. Beyond its effects on lineage allocation, bone marrow adipocytes have been proposed to function as a local reservoir of FFAs for neighboring skeletal cells (54), providing a substrate for oxidative metabolism (20). Notably, marrow adiposity is increased in CKD, potentially reflecting impaired osteoblast FAO and FFA utilization (55, 56). In this context, we find that localized one-time intrafemoral delivery of a low-dose FFA mixture is sufficient to induce osteogenic gene expression in vivo, supporting the concept that microenvironmental FFA availability modulates the osteoblast metabolic state.

From a therapeutic perspective, a critical feature of our study is the uncoupling of FGF23 reduction from hyperphosphatemia in O*-Gpat2^–/–^* mice with CKD. Under physiological conditions, FGF23 lowers serum phosphate primarily by suppressing renal sodium–phosphate reabsorption, with a smaller contribution mediated through reduced active vitamin D and intestinal phosphate absorption (57–59). Consequently, experimental therapeutic strategies that lower FGF23 have been constrained by the predictable rise in serum phosphate; potential improvement of FGF23-driven cardiac hypertrophy was mitigated by hyperphosphatemia, vascular calcification, hypertension, and mortality risk in CKD-MBD (16). In contrast, osteoblast-specific *Gpat2* deletion reduced both FGF23 and serum phosphate despite diminished phosphaturia, both in adenine diet–induced and genetic models of CKD. Although we did not directly assess intestinal phosphate handling, its contribution is largely vitamin D dependent, which was unchanged in our model. Rather, our data support a mechanism in which restoration of osteoblast FAO enhances skeletal phosphate incorporation, thereby increasing bone buffering capacity and reshaping systemic phosphate balance. Enhanced osteoblast mineralization may therefore redirect phosphate toward physiologic skeletal deposition and away from pathologic soft tissue and vascular calcification by reducing the extracellular phosphate burden that drives osteogenic conversion within the vasculature. Both reduced vascular calcification and lower FGF23 levels likely contribute to the improvement in cardiac hypertrophy; future studies are needed to define their relative contributions. Altered phosphate/PTH/FGF23 feedback may further contribute to this phenotype. Increased skeletal phosphate incorporation may lower circulating phosphate early in disease progression, thereby reducing PTH stimulation. Lower PTH levels could subsequently contribute to reduced FGF23 production and decreased bone resorption. Consistent with this interpretation, we observed lower PTH levels at earlier time points throughout the CKD model in O*-Gpat2^–/–^* mice. Notably, this phenotype differs from *Fgf23*-knockout models, which display impaired bone quality and fail to improve bone volume or mineralization in CKD (60). It also contrasts with high-fat feeding in CKD, where FGF23 and phosphate rise in parallel, yet phosphaturic signaling remains functionally uncoupled from effective phosphate lowering (61). Together, these findings reinforce the concept that correcting cell-intrinsic metabolic constraints in bone can recalibrate endocrine mineral regulation and uncouple FGF23 reduction from the hyperphosphatemia that has limited prior therapeutic approaches.

Unlike GPAT1, which plays a major role in hepatic and adipocyte triglyceride synthesis, GPAT2 plays a minor role in systemic lipid metabolism and is primarily restricted to bone and to functions in testicular spermatogenesis (62–64). Consistent with this, manipulation of GPAT2 in bone did not alter systemic lipid profiles in our studies. However, given the essential role of GPAT2 in spermatogenesis, bone-specific delivery is required (62, 63). To address this, we employed a bone-targeted GPAT inhibitor conjugated to poly–aspartic acid, which preferentially localizes to the mineralized matrix. Although this strategy effectively modulated GPAT activity in bone, it lacks isoform specificity. While GPAT2 is the predominant GPAT isoform expressed in bone at baseline, its deletion induced compensatory upregulation of other GPATs, including *Gpat1* in bone marrow adipocytes. Future work should focus on the development of GPAT2-selective inhibitors coupled to optimized bone-targeting strategies, such as poly–aspartic acid or bisphosphonate conjugates, to maximize specificity and safety.

In interpreting our data, we considered potential off-target effects of *Osx*-Cre, which has been reported to target subsets of adipocytes derived from *Osterix-*expressing progenitors (65). However, our expression analyses indicate that *Gpat1* — not *Gpat2* — is the predominant GPAT isoform in bone marrow adipocytes, with further upregulation observed following osteoblast *Gpat2* deletion. In addition, as vascular calcification progresses, cells within calcified lesions may acquire osteogenic features and express *Osterix*, potentially allowing late-stage *Gpat2* deletion within the vasculature. Such effects would likely be restricted to newly differentiated cells and are unlikely to account for the systemic phenotypes observed, but future lineage-tracing studies could be used to resolve this issue.

In summary, our study identifies osteoblast GPAT2 as a previously unrecognized metabolic checkpoint that integrates FFA utilization with skeletal phosphate buffering to shape systemic mineral balance in CKD. By diverting FFAs away from mitochondrial oxidation, GPAT2 limits osteoblast mineralization and amplifies the vascular and cardiac sequelae of phosphate excess. Targeted inhibition of GPAT2 restores osteoblast oxidative capacity, enhances phosphate incorporation into bone, and lowers circulating FGF23 without provoking hyperphosphatemia, overcoming a key limitation of prior therapeutic approaches. These findings reposition bone as an active metabolic regulator rather than a passive target of mineral dysregulation and highlight osteoblast FFA partitioning as a disease-modifying axis in CKD-MBD. Bone-targeted GPAT2 modulation thus offers a promising strategy to simultaneously improve skeletal integrity, vascular health, and cardiac remodeling.

## Methods

### Sex as a biological variable.

Our study employed both male and female mice, with the exception of *Col4a3^–/–^* mice, which were all male because male mice develop a more pronounced kidney phenotype than female mice. Similar findings are reported for both sexes.

### Mice.

C57Bl/6J mice were purchased from The Jackson Laboratory. O-*Gpat2^–/–^* mice were generated by breeding Osx-Cre mice (66, 67) with floxed *Gpat2* mice. Floxed *Gpat2* mice were generated by Sanger Institute (ID: 9106) carrying loxP sequences flanking exon 2 of the *Gpat2* gene (European Mouse Mutant Archive strain number EM:09015, C57BL/6N-A<tm1Brd> Gpat2<tm2a(KOMP)Wtsi>/Wtsi) (Figure 1B). These mice were used as controls for the O-*Gpat2^–/–^* mice. To generate O-*Gpat2^–/–^* mice on a CKD genetic background, we backcrossed them to mice carrying the Alport mutation 129-Col4a3<tm1Dec>/J (The Jackson Laboratory, 002908), which develops CKD-MBD by 14 weeks of age in a mixed background (68). Namely, we intercrossed O-*Gpat2*^fl/fl^ *Osx*-cre^–^ and O-*Gpat2*^fl/fl^ *Osx*-cre^+^ mice with *Col4a3^–/–^* mice. In all cohorts, we further intercrossed the F1 heterozygotes to generate incipient congenic strains.

### Animal experiments.

G-3-P (300 mg/kg; MilliporeSigma), LPA (50 mg/kg; Santa Cruz Biotechnology), and the bone-targeting GPAT inhibitor (0.25–10 mg/kg) were administered via i.p. injection into groups of 3–10 mice.

O-*Gpat2^–/–^* and O-*Gpat2*^+/+^ littermates (12 weeks old, both sexes) were placed on a 0.2% adenine diet (0.9% phosphorus, 0.6% calcium; Teklad, 130900) for 8 weeks to induce CKD. In a separate experiment, mice were placed on one of the following diets to assess cardiovascular phenotype: adenine diet (0.2% adenine, 0.9% phosphorus, 0.6% calcium), adenine diet with high phosphate (0.2% adenine, 2% phosphorus, 0.6% calcium; Teklad, 230714), high-phosphate diet (2% phosphorus, 0.6% calcium; Teklad, 220178), or regular diet (0.9% phosphorus, 0.6% calcium) (69–71). Diets were maintained for 12 weeks to allow development of CKD-MBD. Blood was collected every 2 weeks, and urine and organs were collected at study termination. O-*Gpat2^–/–^* and O-*Gpat2*^+/+^ mice with or without the Alport mutation (*n* = 6 per group) were euthanized at 12 weeks of age, and blood, urine, and organs were collected for analysis.

### Intrafemoral injection.

Intrafemoral injections were performed after 4 weeks of adenine or control diet. Following shaving and skin sterilization, a 26-gauge needle was inserted through the patellar tendon between the femoral condyles at a 90° angle to the bone end face. Using a slow twisting or drilling motion, the needle was advanced until a drop in resistance indicated entry into the marrow cavity (72). An FFA suspension (10 μM linoleic acid, palmitic acid, stearic acid, and PUFA mix, all albumin-bound; Cayman) or vehicle was injected slowly. The needle was withdrawn carefully while maintaining gentle pressure on the injection site.

### Kidney function and mineral metabolism assays.

Creatinine was measured from mouse plasma and urine by liquid chromatography–mass spectrometry (LC-MS) as previously described (28). Phosphate concentrations in plasma, urine, and thoracic aortic tissue digested in EDTA (5 mg/mL) were measured using a colorimetric assay (Abcam). Calcium concentrations in plasma and EDTA-digested aortic tissue were measured using a colorimetric assay (Stanbio). Plasma 1,25-dihydroxyvitamin D_3_ levels were quantified by enzymatic immunoassay (Immunodiagnostic Systems; AC-62F1). Intact FGF23 and PTH were measured by the respective ELISAs (FGF23 mouse/rat, 60–6,800; PTH mouse, 60–2,305; rat, 60–2,500; Immunotopics) according to the manufacturer’s instructions.

Fractional excretion of phosphate was calculated as (urine phosphate × serum creatinine)/(serum phosphate × urine creatinine). Plasma triglycerides were measured using a triglyceride assay kit (Abcam, ab65336) according to the manufacturer’s instructions.

### LC-MS.

G-3-P was measured in 10 μL of mouse plasma after extraction with 90 μL of acetonitrile/methanol (3:1, vol/vol) containing 1 μg/mL G-3-P-^13^C_3_ (Cambridge Isotope Laboratories). Samples were centrifuged, and 10 μL of supernatant was injected onto a 150 × 2.1 mm Atlantis hydrophilic interaction chromatography silica 3 μm column (Waters). The column was eluted at 250 μL/min with 5% mobile phase A (10 mM ammonium formate in water) for 0.5 minutes, followed by a linear gradient to 40% mobile phase B (acetonitrile) over 8 minutes, held at 40% B for 5.5 minutes, and then returned to starting conditions. G-3-P (transition 170.99/78.99) and the isotopic standard G-3-P-^13^C3 (transition 173.99/78.99) were monitored in negative ion mode on a TSQ Quantiva Triple Quadrupole Mass Spectrometer (Thermo Fisher Scientific).

For LPA and FFA quantification in cell culture samples, cells were extracted with 800 μL of 80% methanol per well of a 6-well plate. Extracts from mouse plasma and cell lysates were evaporated and reconstituted in 50 μL of 80% methanol. For LPA measurements, a 10-μL aliquot was separated on a reversed phase column (Acquity UPLC C18 column 2.1 × 75 mm), and LPA 18:1 (transition 435.1/152.9) was detected in negative ion mode on a Q-Exactive Plus Orbitrap mass spectrometer (Thermo Fisher Scientific) with HESI probe and TSQ Quantiva Triple Quadrupole Mass Spectrometer (28, 73).

FFA levels were measured by gas chromatography–mass spectrometry using an 8890B GC coupled to an EI/CI 5977 MSD (Agilent) in chemical ionization mode with methane as a reagent gas and helium as carrier gas as previously described (74). Metabolites were identified by matching retention times and mass-to-charge ratios to synthetic reference standards and characterized pooled reference samples (28).

### Cell culture experiments.

Primary osteoblasts were generated from femoral bone marrow stromal progenitor cells as previously described (75). Briefly, bone marrow cells were isolated from femurs and cultured for 3 weeks to obtain a purified stromal progenitor population. Cells were then differentiated into osteoblasts in osteogenic medium containing 0.1 mM dexamethasone, 50 μM ascorbic acid, and 10% FBS or 10% lipid-reduced serum, produced by mixing FBS with fumed silica (76), supplemented with 1% antibiotic for 2–3 weeks (75). Murine primary bone marrow cells were differentiated into adipocytes by adding dexamethasone (1 μM), indomethacin (0.2 mM), insulin (100 nM), and 3-isobutyl-1-methylxanthine (0.5 mM) (MilliporeSigma) in αMEM with 10% FBS. After 3 days of induction, committed preadipocytes were allowed to mature in αMEM containing 10% FBS and insulin (100 nM).

Primary osteoblasts were treated with 10 μM G-3-P (MilliporeSigma), 10 nM 1,25-dihydroxyvitamin D_3_ (Enzo Life Sciences), an FFA mix (10 μM each of the FFAs used in the in vivo experiments) bound to albumin, etomoxir (MilliporeSigma, 100 μM), or GW9508 (MilliporeSigma,10 nM).

*Cpt2* was knocked down using adeno-associated virus particles targeting *Cpt2* (Origene, NM_009949), *Ffar4* was overexpressed using lentiviral particles (Origene, NM_181748), and GFP was introduced using adenoviral particles (Vector Biolabs, 20230309). Viral packaging was performed in 293T cells (ATCC) according to standard protocols (https://portals.broadinstitute.org/gpp/public/resources/protocols). Primary osteoblasts were exposed to lentiviral particles (MOI = 1) overnight in the presence of polybrene (5 μg/mL), followed by selection with puromycin (2 μg/mL) and maintenance in selection medium for the duration of experiments.

Osteoblast colonies were stained with alkaline phosphatase (MilliporeSigma) to quantify colony-forming units or with alizarin red (MilliporeSigma) or von Kossa to assess mineralization as previously described (75, 77). Mineralized nodules were quantified using ImageJ (NIH).

### Seahorse.

OCRs of primary osteoblasts from O-*Gpat2^–/–^* and O-*Gpat2*^+/+^ mice, with and without CKD, were measured by a Seahorse XFe96 Analyzer (Agilent) according to the manufacturer’s instructions. Bone marrow cells were plated directly into Seahorse assay plates at 6 × 10^5^ cells per well in a 96-well plate after isolation and differentiated into osteoblasts for 3 weeks, as described above.

### Histology, histomorphometry, and immunohistochemistry.

Bone, heart, and kidney tissues were fixed in formalin, embedded in paraffin, and sectioned at 3 μm. Sections were stained with H&E, von Kossa, or alizarin red to assess tissue mineralization (6 sections per organ). For bone histomorphometry, animals received subcutaneous injections of the fluorochrome calcein (10 mg/kg; Sigma-Aldrich) at 4 and 14 days before euthanasia. Femurs were collected, processed for histomorphometric analysis, and quantified using a computer-assisted image analysis system (OsteoMeasure, OsteoMetrics Inc.) as previously described (75). GPAT2 expression was assessed on 3 consecutive bone sections using a GPAT2 antibody (Bioss, bs13497R/AH10087297), followed by detection with an HRP-coupled secondary anti-rabbit antibody (Invitrogen, 65-6120/3355947).

### Immunofluorescence staining.

Cells were fixed in 4% paraformaldehyde in PBS for 15 minutes at room temperature. For colocalization studies, cells were stained with BODIPY C12 and C16 (Invitrogen, D3835 and D3821) for 12 hours. Mitochondria were visualized using MitoView Green (Biotium, 70054) with a 30-minute incubation.

Deparaffinized bone sections were stained with a GPAT2 antibody followed by an FITC-labeled secondary anti-rabbit antibody (Invitrogen, A-11034/1851447). The bone-targeted GPAT inhibitor was conjugated to tetramethylrhodamine (TMR).

### qPCR.

Total RNA was extracted from cultured cells using RNeasy (Qiagen) following the manufacturer’s instructions. For long-bone RNA isolation, mice were euthanized, and femurs were rapidly dissected on ice. Soft tissue was removed and epiphyses cut. Bone marrow cells were then removed by serial flushing with ice-cold PBS. TRIzol (Life Technologies, Thermo Fisher Scientific) was added, and samples were frozen at –80°C and then homogenized. For bone marrow RNA, flushed bone marrow was spun down and homogenized with TRIzol. RNA was then extracted according to the manufacturer’s instructions. For cDNA synthesis, 1 μg RNA was used for the synthesis reactions according to the manufacturer’s instructions (PrimeScript RT; Takara). SYBR Green–based qPCR detection was performed using FastStart Universal SYBR Green (Roche) on a StepOne Plus Thermocycler (Applied Biosystems). The PCR primer sequences are listed in Supplemental Table 3.

### Immunoblotting.

Whole-cell lysates were prepared using RIPA buffer with protease inhibitors (Pierce, Thermo Fisher Scientific). Lysates (15–20 μg cellular protein) were separated by SDS-PAGE, and proteins were transferred onto nitrocellulose membranes. Membranes were blocked with 5% milk in TBST and incubated with FFAR4 (Abcam, ab263964/1013193-4), CPT2 (Invitrogen, A21974/3184497), GPAT2 (Bioss, bs13497R/AH10087297), or GAPDH (Abcam, ab8245/1090835-2) antibodies overnight at 4°C. The next day, the membranes were washed and incubated with the appropriate HRP-coupled secondary antibodies, and signals were detected with ECL (Pierce, Thermo Fisher Scientific). All immunoblots were repeated at least twice, with comparable results.

### Cardiac ultrasound.

Echocardiography was performed in unanesthetized mice using a GE E90 ultrasound system with a high-frequency L8-18i-D transducer. Parasternal short- and long-axis views were acquired for 2D and M-mode imaging. Relative wall thickness was calculated as (interventricular septum diastolic [IVSd] + left ventricular posterior wall diastolic)/left ventricular diastolic diameter (11).

### Micro-CT.

Imaging analyses were performed at the MGH Center for Skeletal Research Imaging on distal femurs using a high-resolution desktop microtomographic imaging system (μCT40, Scanco Medical AG) by a blinded investigator. Scans were acquired with a 10 μm^3^ isotropic voxel size, 70 kVp, 114 μA, and 200 ms integration time and were subjected to Gaussian filtration and segmentation. In the distal femur, transverse micro-CT slices were evaluated beginning 200 μm superior to the distal growth plate and extending proximally 1.5 mm. The trabecular bone regions were identified by semimanually contouring the endocortical region of the bone, with a threshold of 400 mgHA/cm^3^ to segment bone from soft tissue. Cortical bone was analyzed across 0.5 mm at the femoral mid-diaphysis, including the entire outermost edge of the cortex. Image acquisition and analysis protocols adhered to published guidelines (78).

### Synthesis of bone-targeted GPAT inhibitor.

The bone-targeted GPAT inhibitor FSG67-K(TMR)-(Asp)_6_-amide was synthesized using standard Fmoc/tBu solid-phase peptide chemistry on Rink Amide MBHA resin (Akaal Organics) (100 μmol scale) (79). Six aspartic acid residues were sequentially coupled using microwave-assisted synthesis, followed by manual incorporation of an N-terminal lysine [Fmoc-Lys(Dde)-OH]. The GPAT inhibitor FSG67 was coupled to the peptide N-terminus, and TMR was conjugated to the lysine side chain after Dde deprotection. The peptide was cleaved and globally deprotected using Reagent K (80), precipitated in cold methyl tert-butyl ether, and purified by reverse-phase HPLC on a C18 column. Product identity and molecular weight were confirmed by MALDI-MS.

### Human bone samples.

Bone samples were obtained from pediatric patients with end-stage renal disease undergoing chronic cycling peritoneal dialysis who received trans-iliac bone biopsies at UCLA between January 2008 and March 2011 (*n* = 6) (14). Control samples were obtained from the UCLA bone tissue bank (*n* = 3). After removal of muscle and cortical bone, biopsy cores were minced, washed in antibiotic-antimycotic media (Gibco), and plated for adherence (37°C, 5% CO_2_). Cells were cultured in DMEM containing 20% FBS and ascorbic acid (100 μg/mL), with media changed twice weekly. After colony formation (1–4 weeks), cells were pooled, expanded, and cryopreserved at approximately 80% confluence in 10% DMSO/30% FBS for future use.

### Statistics.

Statistical analyses were performed using GraphPad Prism 10 (GraphPad Software). Sample size (*n*) refers to biologically independent replicates and is indicated in the figure legends. Data are presented as mean ± SEM unless otherwise stated. Comparisons between 2 groups were conducted using Welch’s *t* test. For multiple pairwise comparisons, Welch’s *t* test with Bonferroni’s correction was applied. Two-way ANOVA was used for experiments involving 2 independent variables. Statistical details for each experiment are provided in the figure legends. All *t* tests were 2 sided, and *P* < 0.05 was considered statistically significant.

### Study approval.

Bone biopsy samples were collected from patients who participated in previously approved institutional studies. All participants provided written informed consent to have their samples stored for future use. The institutional ethics committee of UCLA approved this project. All animal studies in the present work were approved by the IACUC of Massachusetts General Hospital.

### Data availability.

Values for all data points in graphs are reported in the Supporting Data Values file.

## Manuscript information

### Author contributions.

Conceptualization: PS and EPR. Methodology: PS, HX, WZ, YF, RCP, FD, JDR, IBS, RPG CS, and AK. Formal analysis: PS, IBS, and EPR. Investigation: PS, HX, WZ, YF, RCP, FD, JDR, IBS, RS, CS, and AK. Resources: PS and EPR. Writing (original draft): PS. Writing (review and editing): EPR and PS with input from all authors.

### AI disclosure.

ChatGPT Model GPT-6 was utilized solely for copyediting, grammatical corrections, and language refinement during the drafting of the manuscript from February 1, 2026 through October1, 2026. The final text was thoroughly reviewed, verified, and approved by all authors.

### Conflict of interest.

AK, EPR, and PS have US patent application number 63/826,497. WZ is employed by Johnson & Johnson. JDR has received research support from Amgen and Keros Therpaeutics.

### Funding.

This study was supported by the following:

- National Institute of Diabetes and Digestive and Kidney Diseases (NIDDK) K08DK124568 and R01DK146964 (to PS).
- Massachusetts General Hospital Transformative Scholar Award (to PS).
- Massachusetts General Hospital Executive Committee on Research and Center for Skeletal Research Pilot Grant (to PS).
- National Heart, Lung, and Blood Institute grant R01HL170048 (to JDR).
- American Heart Association grant 25SFRNPCKMS463897 (to JDR).
- Yeatts Fund for Innovative Research (to JDR).
- NIDDK grants R01DK35423 and U01DK122013 (to IBS).
- Burroughs Wellcome Fund Career Award for Medical Scientists (to RPG).
- NIDDK grant R01DK134675 (to RPG).
- NIDDK grant R01DK130291 (to EPR).

This work is the result of NIH funding, in whole or in part, and is subject to the NIH Public Access Policy. Through acceptance of this federal funding, the NIH has been given a right to make the work publicly available in PubMed Central.

## Supplementary Material

Supplemental data

Unedited blot and gel images

Supporting data values

## Figures and Tables

**Figure 1:**
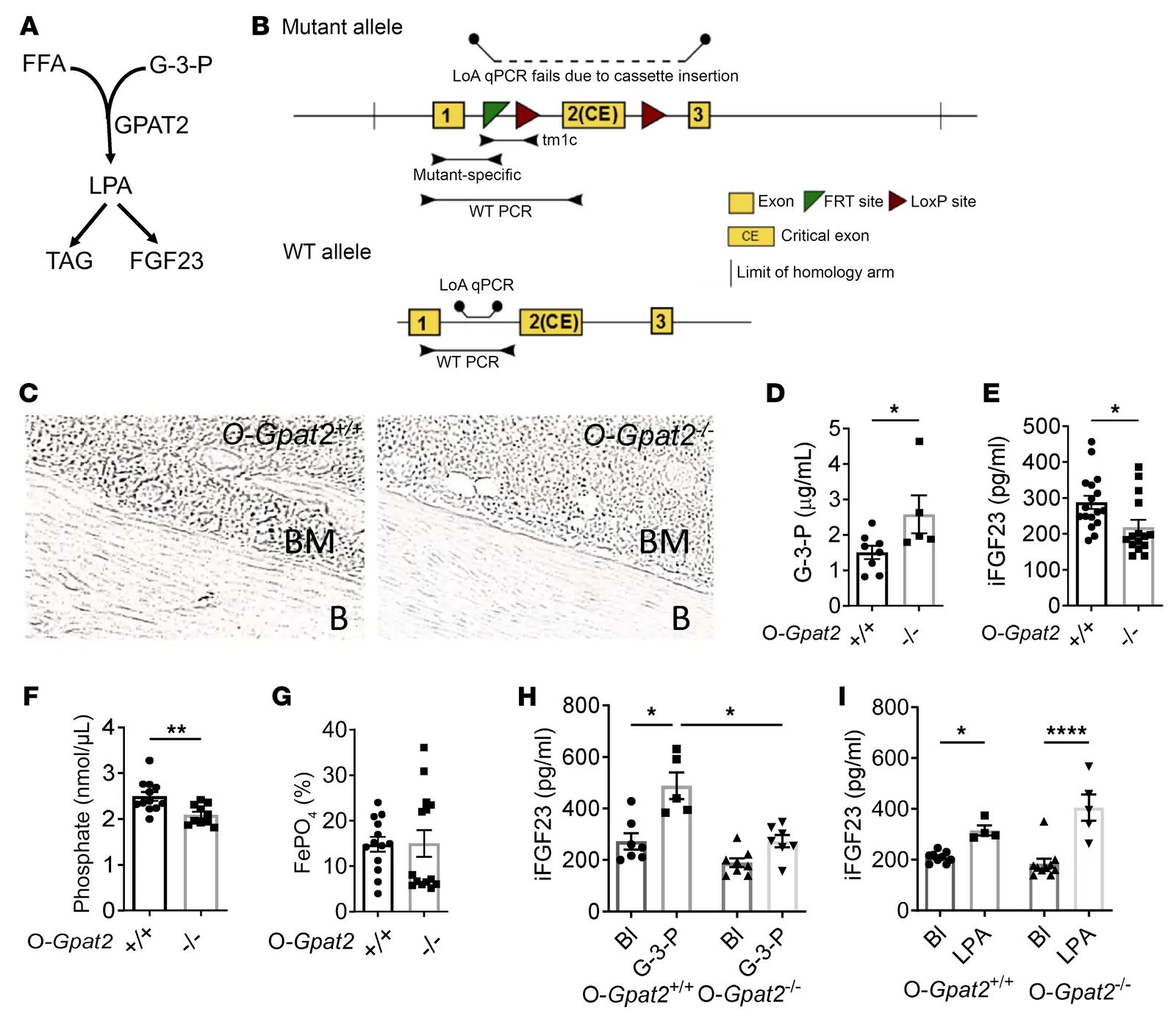
Osteoblast *Gpat2* regulates FGF23 production and blood phosphate levels. (**A**) Schematic of the GPAT2-mediated reaction. (**B**) Schematic of the WT and *Gpat2* mutant alleles. LPA, lysophosphatidic acid;TAG triacylglycerol. (**C**) Immunohistochemistry for GPAT2 in femoral bone from O-*Gpat2*^+/+^ and O-*Gpat2*^–/–^ mice (original magnification, ×20). B, bone; BM, bone marrow. (**D**–**G**) Plasma G-3-P (**D**), intact FGF23 (iFGF23) (**E**), plasma phosphate (**F**), and urinary fractional excretion of phosphate (FePO_4_) (**G**) in O-*Gpat2*^+/+^ and O-*Gpat2*^–/–^ mice. (**H** and **I**) Plasma iFGF23 at baseline (Bl) and 24 hours after administration of G-3-P (**H**) or LPA (**I**) in O-*Gpat2*^+/+^ and O-*Gpat2*^–/–^ mice. Data are mean ± SEM. Statistical significance was assessed by Welch’s *t* test (**D**–**G**) or 2-way ANOVA (**H** and **I**). *n* = 5–17 mice per group, male and female, 12 weeks of age. *P < 0.05, **P < 0.01, ****P < 0.001.

**Figure 2:**
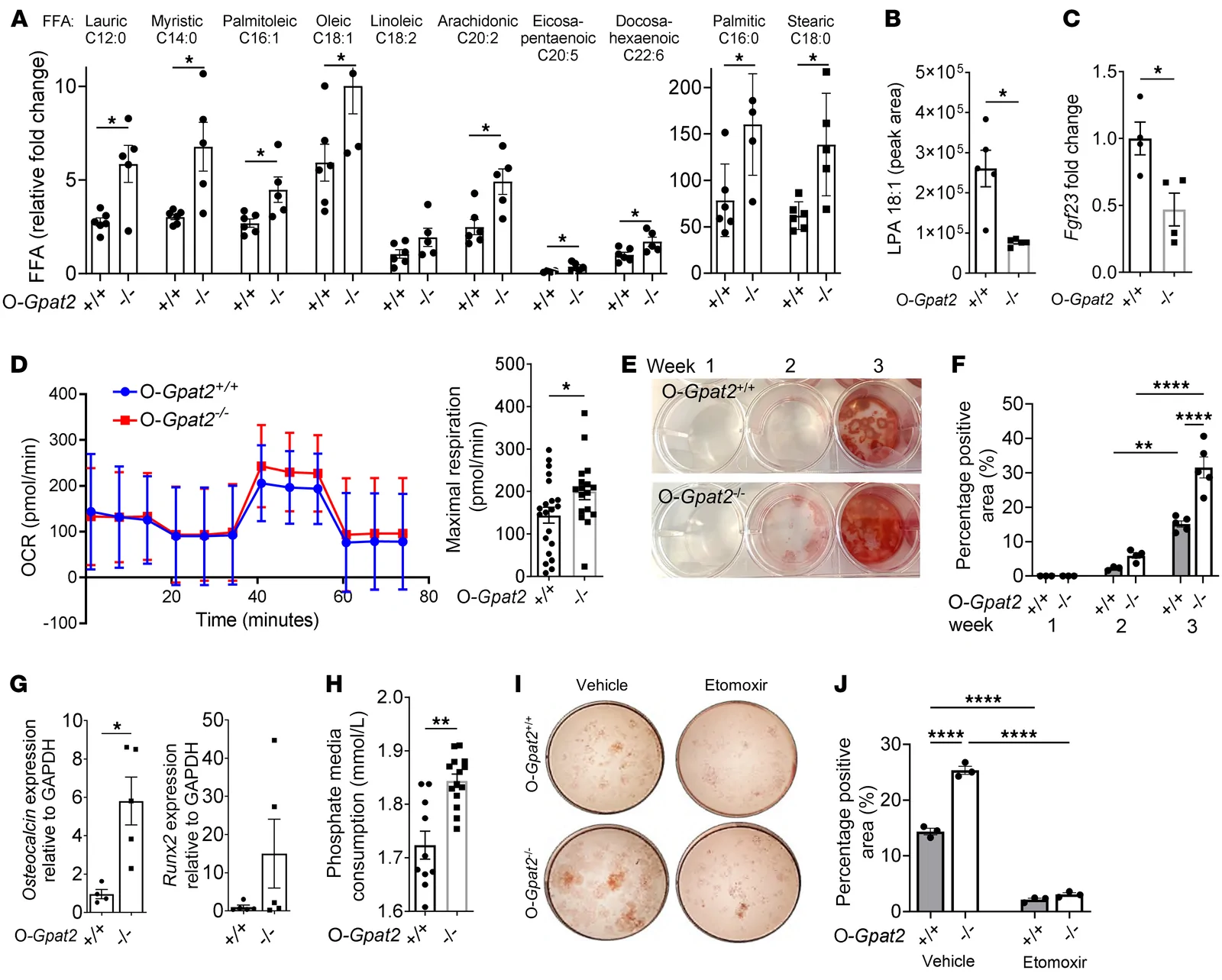
GPAT2 limits osteoblast FFA availability, oxidative metabolism, and mineralization. (**A**–**C**) FFA (**A**) and LPA (**B**) levels and *Fgf23* expression (**C**) relative to GAPDH in primary osteoblasts from O-*Gpat2*^+/+^ (+/+) and O-*Gpat2*^–/–^ (–/–) mice. (**D**) OCR (left) and maximal respiration (right) in primary osteoblasts from O-*Gpat2*^+/+^ and O-*Gpat2*^–/–^ mice. (**E** and **F**) Alizarin red staining across weeks of osteogenic differentiation (**E**) and corresponding ImageJ quantification (**F**). (**G** and **H**) Osteogenic gene expression measured by qPCR (**G**) and phosphate consumption from culture media (**H**) after 3 weeks of differentiation of primary osteoblasts from O-*Gpat2*^+/+^ and O-*Gpat2*^–/–^ mice. (**I** and **J**) Representative alizarin red staining of osteoblasts from O-*Gpat2*^+/+^ and O-*Gpat2*^–/–^ mice treated with etomoxir (100 μM) during differentiation (**I**) and ImageJ quantification of alizarin red^+^ area (**J**). Vehicle-treated O-*Gpat2*^+/+^ and O-*Gpat2*^–/–^ primary osteoblasts are shared controls for the experiment shown in Figure 6H, and statistical analysis of the quantification accounts for all comparisons. Primary osteoblasts were differentiated from bone marrow stromal cells and analyzed after 3 weeks unless otherwise indicated. Data are mean ± SEM. Statistical significance was assessed by Welch’s *t* test with Bonferroni’s correction (**A**), Welch’s *t* test (**B**–**D**, **G**, and **H**), or 2-way ANOVA (**F** and **J**). *n* = 3–8 wells per group. *P < 0.05, **P < 0.01, ****P < 0.0001.

**Figure 3:**
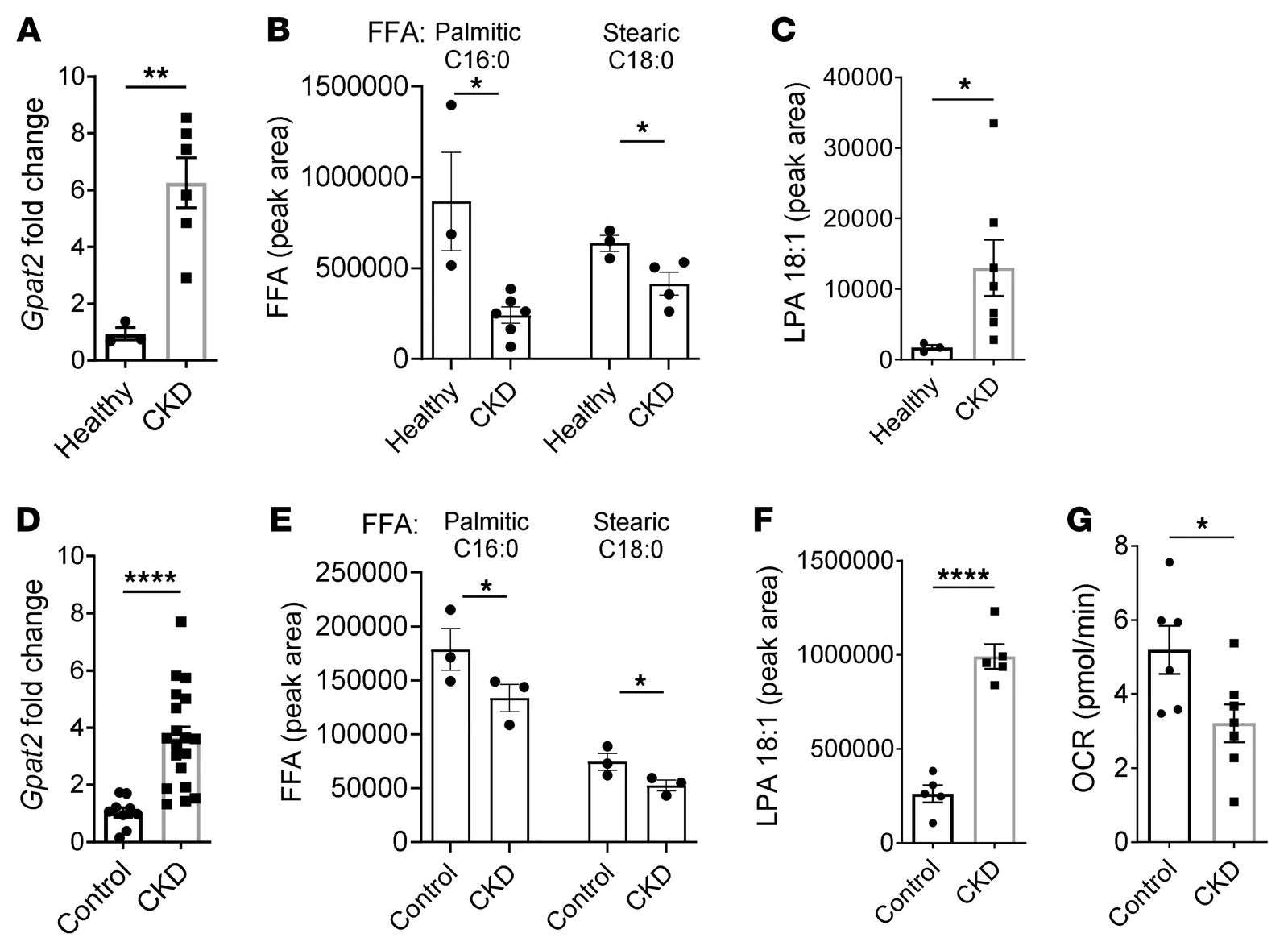
CKD increases GPAT2 expression and activity in mouse and human osteoblasts. (**A**–**C**) *GPAT2* expression normalized to GAPDH (**A**), intracellular FFAs (**B**), and LPA levels (**C**) in primary osteoblasts from patients with CKD. (**D**–**G**) *Gpat2* expression relative to Gapdh (**D**), FFAs (**E**), LPA levels (**F**), and OCR (**G**) in primary osteoblasts isolated from control and adenine diet–induced CKD mice (4 weeks). Data are mean ± SEM. Statistical significance was assessed by Welch’s *t* test (**A**, **C**, **D**, **F**, and **G**) or Welch’s *t* test with Bonferroni’s correction (**B** and **E**). Human samples: healthy *n* = 3, CKD *n* = 6; mouse samples *n* = 3–18 per group. *P < 0.05, **P < 0.01, ****P < 0.0001.

**Figure 4:**
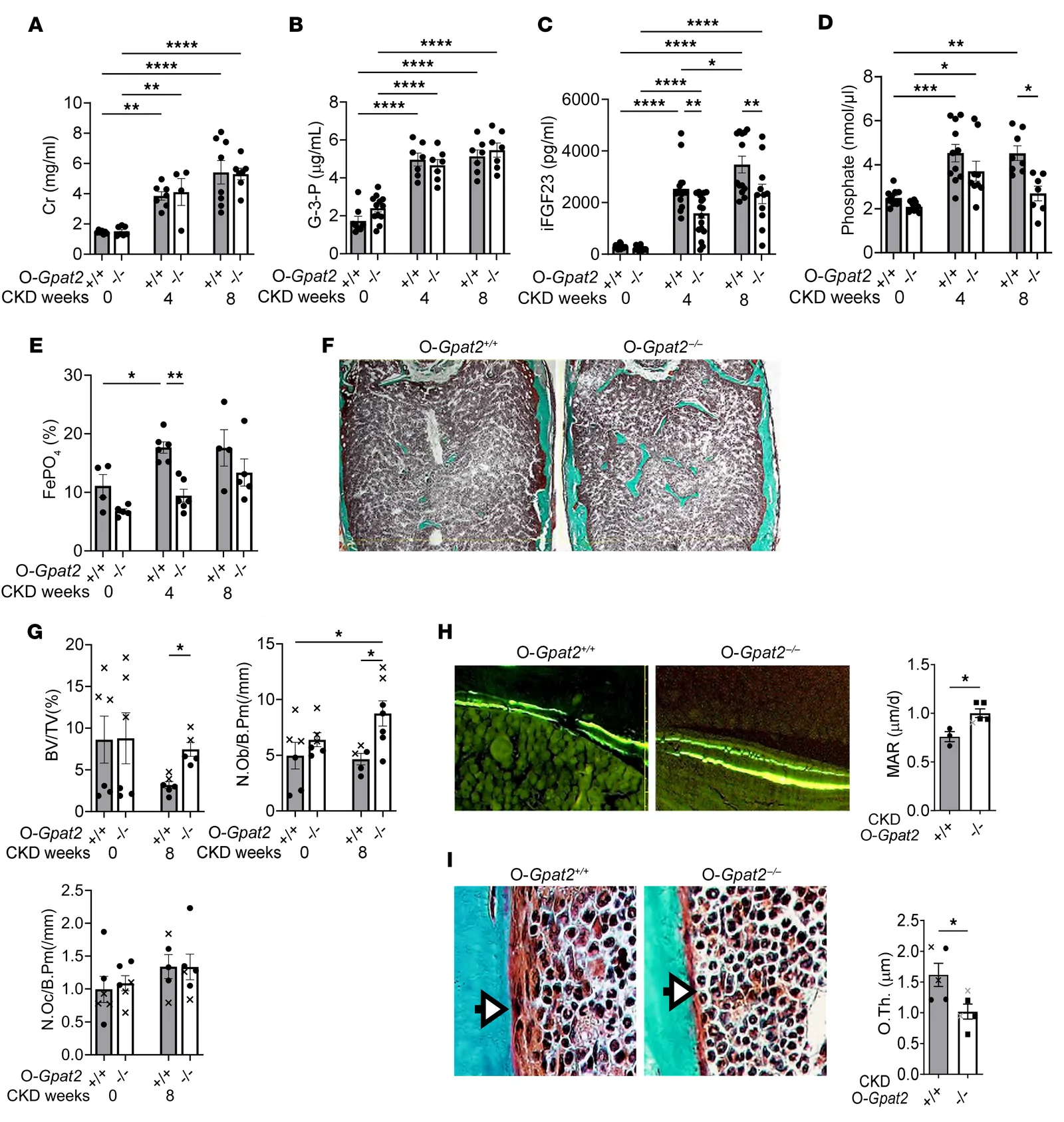
Osteoblast-specific *Gpat2* deletion preserves mineral and bone metabolism in CKD. (**A**–**E**) Plasma creatinine (**A**), G-3-P (**B**), iFGF23 (**C**), phosphate (**D**), and urinary fractional excretion of phosphate (FePO_4_) (**E**) in O-*Gpat2*^+/+^ and O-*Gpat2*^–/–^ mice after 8 weeks of adenine diet–induced CKD. (**F** and **G**) Masson’s trichrome staining of distal femur sections (**F**) and histomorphometric analysis (**G**). (**H**) Double calcein labeling of endosteal cortical bone (left) and mineral apposition rate (MAR; right). (**I**) Endosteal cortical osteoid staining (left) and osteoid thickness (right) in O-*Gpat2*^+/+^ and O-*Gpat2*^–/–^ mice after 8 weeks of adenine diet–induced CKD. Original magnification, ×5 (**F**), ×20 (**H** and **I**). BV/TV, bone volume/trabecular volume; N.Ob/B.Pm, osteoblast number/bone perimeter; N.Oc/B.Pm, osteoclast number/bone perimeter. Data are mean ± SEM. Statistical significance was determined by 2-way ANOVA (**A**, **C**, **D**, and **G**) or Welch’s *t* test (**B**, **E**, **H**, and **I**). *n* = 4–12 mice per group, male (X) and female (circle), 12–20 weeks of age. *P < 0.05, **P < 0.01, ***P < 0.001, ****P < 0.0001.

**Figure 5:**
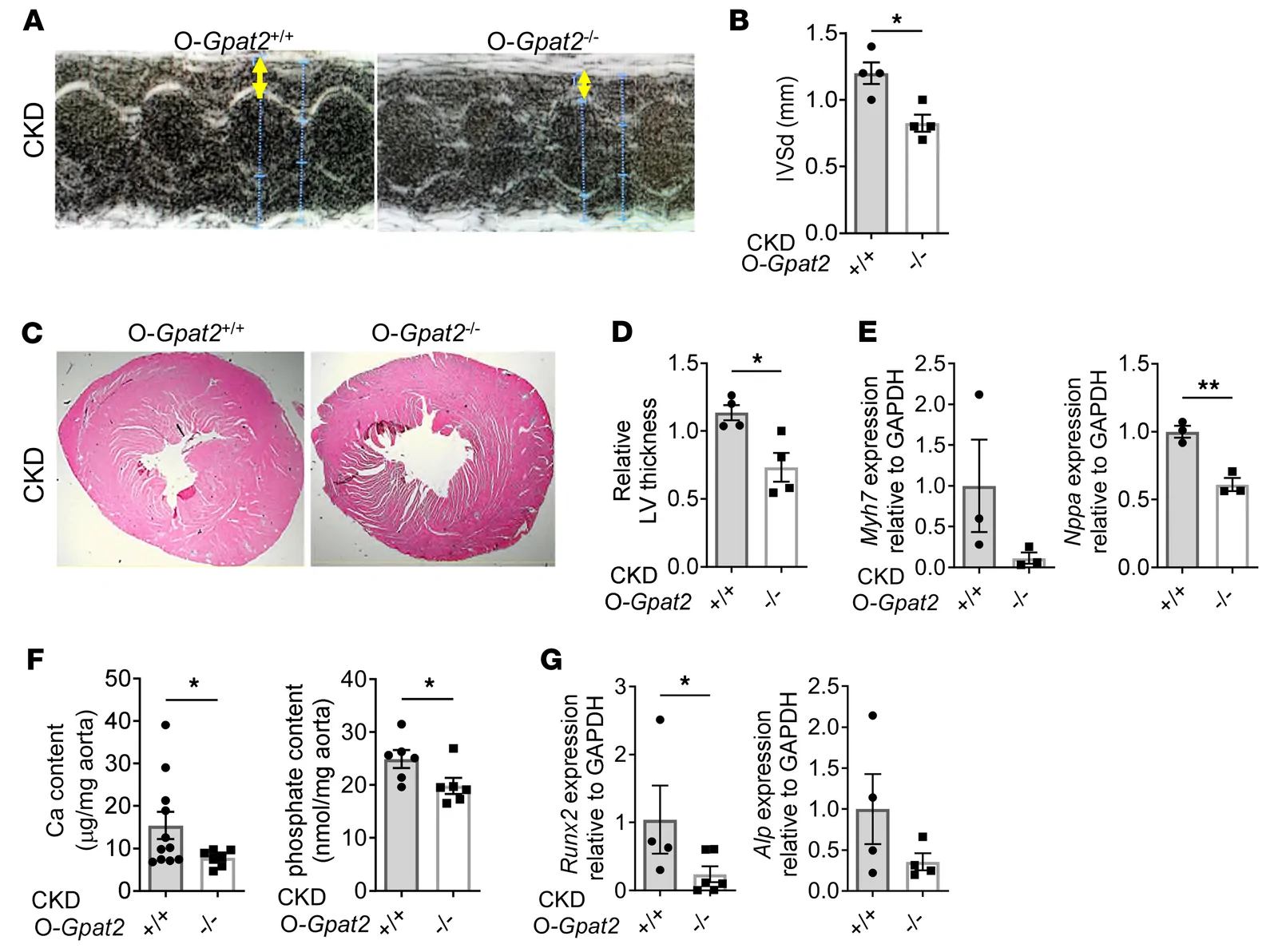
Osteoblast *Gpat2* deletion protects against CKD-associated cardiac hypertrophy and vascular calcification. (**A**) Representative M-mode echocardiography images (yellow arrows indicate interventricular septal diameter, [IVSd]). (**B**) Quantification of IVSd. (**C**–**E**) Representative images of H&E staining of left ventricular cross sections (×5) (**C**), relative left ventricular (LV) wall thickness (**D**), and qPCR analysis of cardiac hypertrophy markers (*Myh7* and *Nppa*) (**E**) from O-*Gpat2*^+/+^ and O-*Gpat2*^–/–^ mice after 12 weeks of adenine diet–induced CKD. (**F** and **G**) Vascular calcium content (**F**) and expression of osteogenic genes (*Runx2* and *Alp*) (**G**) in aortas of O-*Gpat2*^+/+^ and O-*Gpat2*^–/–^ mice after 12 weeks of adenine diet– and high phosphate–induced CKD. Data are mean ± SEM. Statistical significance was determined by Welch’s *t* test. *n* = 3–12 mice per group, male and female, 20–24 weeks of age. *P < 0.05, **P < 0.01.

**Figure 6:**
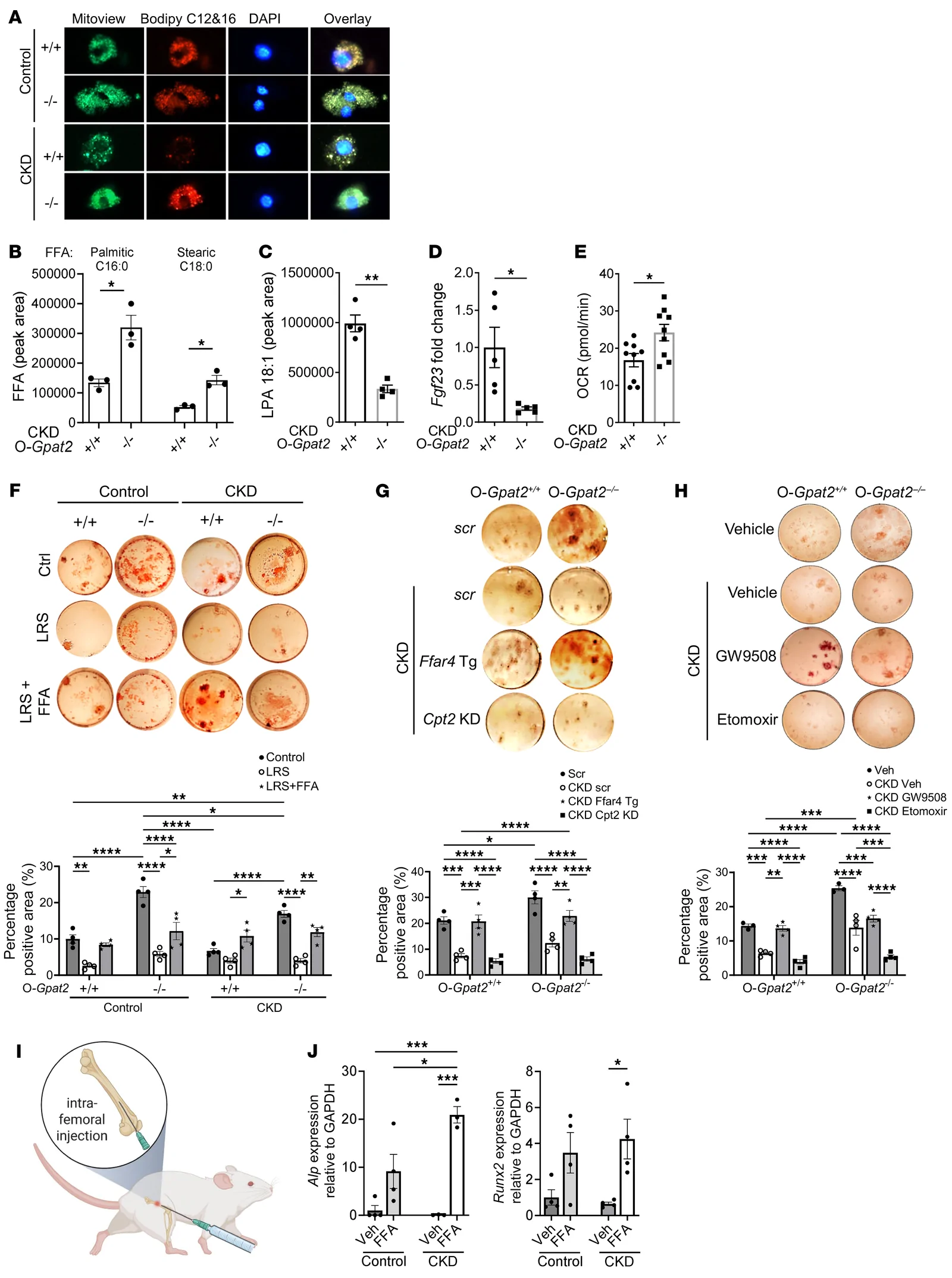
Gpat2 deficiency increases mineralization by increasing osteoblast FAO. (**A**) Representative images of primary osteoblasts from O-*Gpat2*^+/+^ and O-*Gpat2*^–/–^ mice after 4 weeks of adenine diet–induced CKD or control diet stained with MitoView (mitochondria), BODIPY-C12/16 (FFAs), and DAPI (nuclei). Original magnification, ×40. (**B**–**E**) Intracellular FFA levels (**B**), LPA 18:1 abundance (**C**), *Fgf23* expression relative to GAPDH (**D**), and OCR (**E**) in primary osteoblasts from O-*Gpat2*^+/+^ and O-*Gpat2*^–/–^ mice after 4 weeks of adenine diet–induced CKD. (**F**) Alizarin red staining after 3 weeks of osteogenic differentiation in lipid-reduced serum (LRS) with or without FFA supplementation. Vehicle-treated O-*Gpat2*^+/+^ and O-*Gpat2*^–/–^ primary osteoblasts are shared controls for the experiment shown in Figure 2, I and J, and statistical analysis of the quantification accounts for all comparisons. (**G** and **H**) Mineralization after *Ffar4* overexpression (*Ffar4*-Tg) or *Cpt2* knockdown (*Cpt2*-KD) (**G**) and following treatment with the FFA receptor agonist GW9508 or the FAO inhibitor etomoxir (**H**) in primary osteoblasts from O-*Gpat2*^+/+^ and O-*Gpat2*^–/–^ mice after 4 weeks of adenine diet–induced CKD or control diet, following 3 weeks of osteogenic differentiation. Quantification of alizarin red^+^ area by ImageJ is shown below each panel. (**I** and **J**) Schematic of intrafemoral FFA delivery, with vehicle to contralateral femur (**I**), and qPCR analysis of osteogenic gene expression (**J**) 24 hours after injection in mice after 4 weeks of adenine diet–induced CKD or control diet. Data are mean ± SEM. Statistical significance was determined by 2-way ANOVA (**F**–**H**), 1-way ANOVA (**J**), and Welch’s *t* test (**B**–**E**). *n* = 3–4 per group. *P < 0.05, **P < 0.01, ***P < 0.001, ****P < 0.0001.

**Figure 7:**
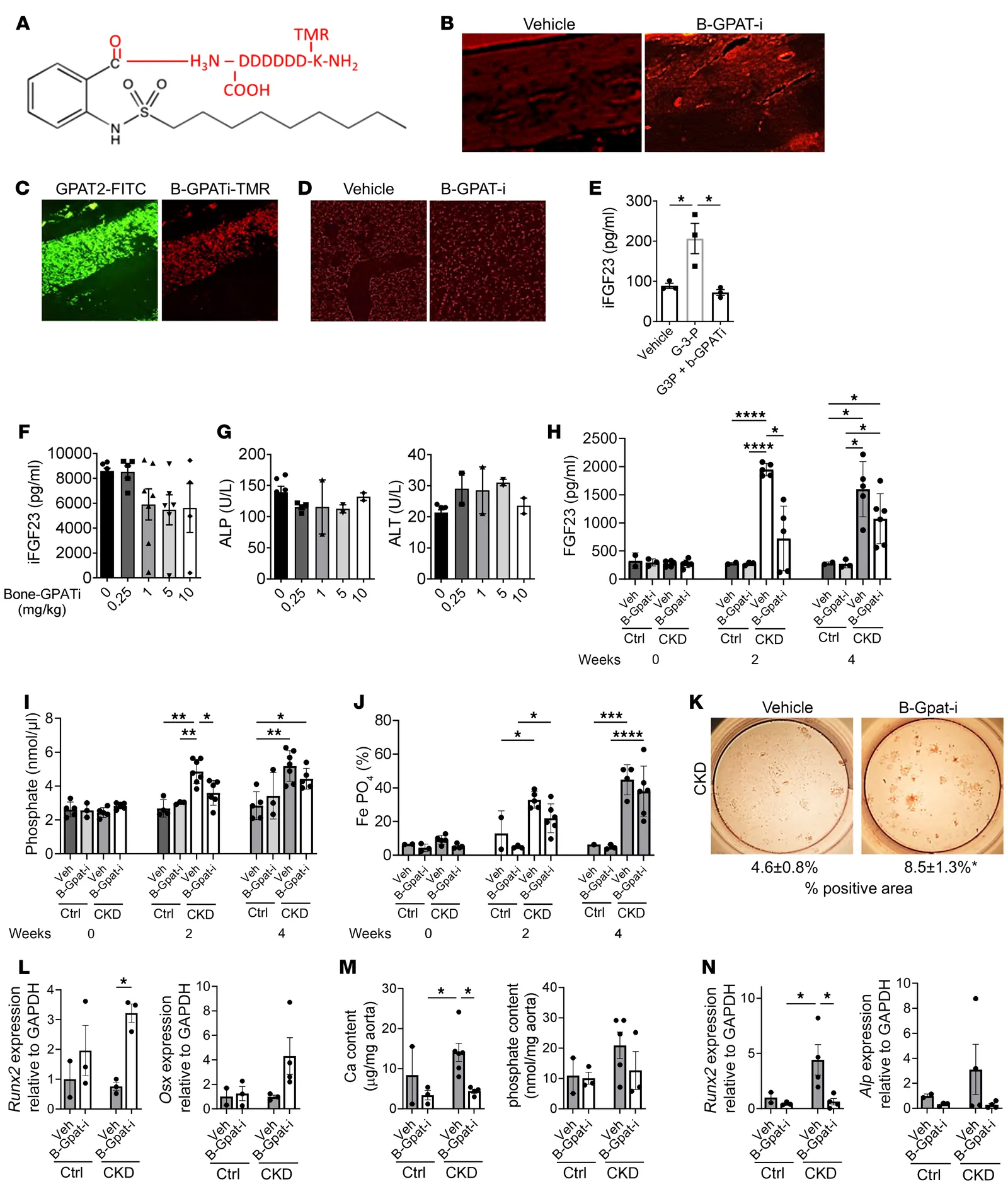
Bone-targeted GPAT inhibition preserves mineral homeostasis in CKD. (**A**) Structure of the bone-targeted GPAT inhibitor conjugated to 6 aspartate residues and tetramethylrhodamine (TMR). (**B**–**D**) TMR fluorescence in the distal femur (**B**), immunofluorescence showing GPAT2 (FITC, green) and bone-targeted GPAT inhibitor (B-GPATi; TMR, red) localization in the distal femur (**C**), and TMR fluorescence in liver (**D**) 24 hours after a single dose of B-GPATi (5 mg/kg) to a WT mouse. (**E**) Plasma intact FGF23 (iFGF23) in G-3-P–challenged mice 24 hours after b-GPATi treatment. (**F** and **G**) Plasma iFGF23 (**F**) and alkaline phosphatase (ALP) and alanine aminotransferase (ALT) (**G**) in mice with adenine-induced CKD (4 weeks) treated with escalating doses of b-GPATi for 24 hours. (**H**–**J**) Plasma iFGF23 (**H**), phosphate (**I**), and fractional excretion of phosphate (FePO_4_) (**J**) in control and adenine diet–induced CKD mice treated with b-GPATi or vehicle for 4 weeks. (**K** and **L**) Alizarin red staining (**K**) and osteogenic gene expression (**L**) in primary osteoblasts from control and adenine diet–induced CKD mice treated with b-GPATi or vehicle for 4 weeks. (**M** and **N**) Vascular calcium and phosphate content (**M**) and osteogenic gene expression (**N**) in thoracic aorta from control and CKD mice treated with b-GPATi or vehicle for 4 weeks. Images are shown at ×20 (**B**) or ×10 (**C** and **D**) magnification. Data are mean ± SEM. Statistical significance was determined by 2-way ANOVA. *n* = 3–6 mice per group. *P < 0.05, **P < 0.01, ***P < 0.001, ****P < 0.0001.

## Version 1 09/29/2026

In-Press Preview

## Version 2 11/16/2026

Electronic publication
