# Supplementary material for: Targeting osteoblast fatty acid metabolism attenuates skeletal and cardiovascular complications in chronic kidney disease models
Source: J Clin Invest. 2026 Sep 29;136(22):e207322. doi: 10.1172/JCI207322 (PMC13767616; doi:10.1172/JCI207322)
Supplement: Supplementary file 1 [file jci-136-207322-s153.pdf]

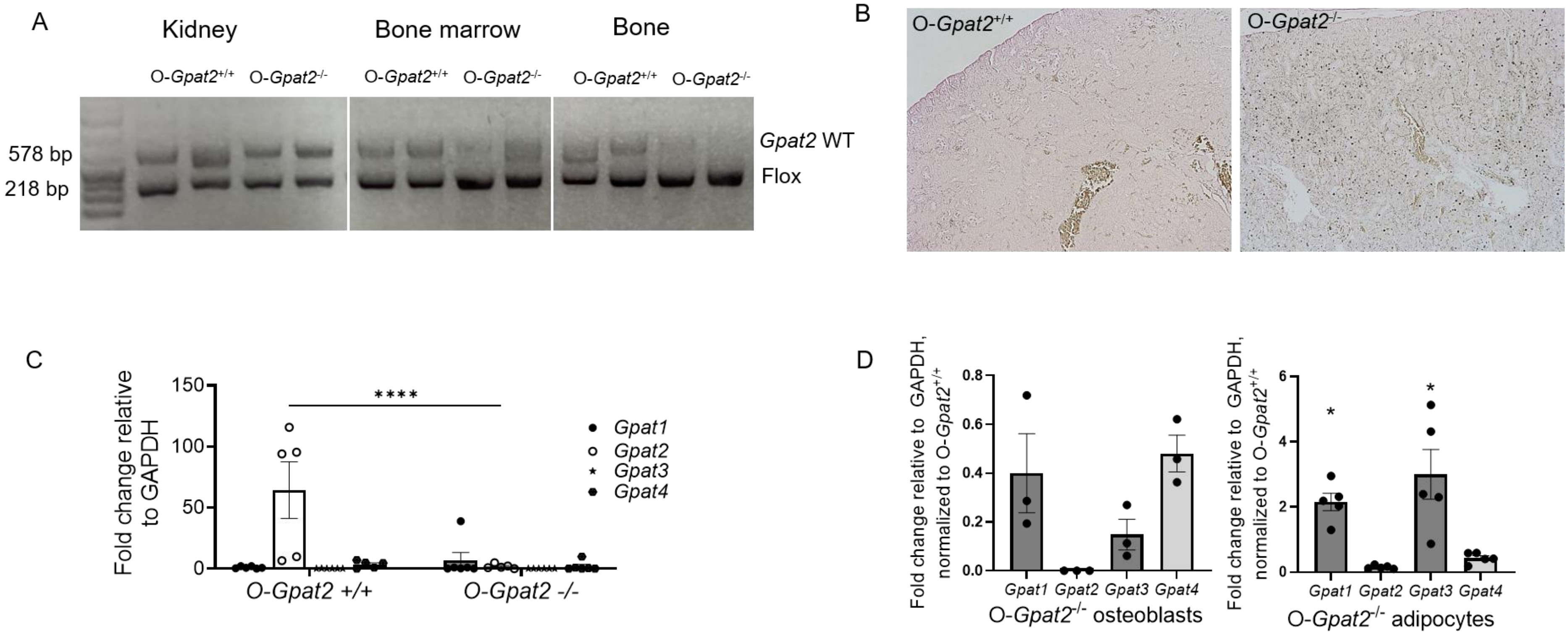

Supplemental Figure 1. Characterization of O-*Gpat2*$^{-/-}$ mice. (**A**) PCR analysis of *Gpat2* expression in kidney, bone marrow, and bone from O-*Gpat2*$^{+/+}$ and O-*Gpat2*$^{-/-}$ mice. (**B**) Immunohistochemistry for GPAT2 in kidney sections from O-*Gpat2*$^{+/+}$ and O-*Gpat2*$^{-/-}$ mice (5× magnification). (**C**, **D**) qPCR analysis of *Gpat1–4* expression in (**C**) bone and (**D**) osteoblasts and adipocytes from O-*Gpat2*$^{+/+}$ and O-*Gpat2*$^{-/-}$ mice. *n* = 3-6 per group, male and female mice, 12 weeks of age. *P* values by two-way ANOVA (**C**) and Welch's *t*-test (**D**). * $p<0.05$, ****$p<0.0$

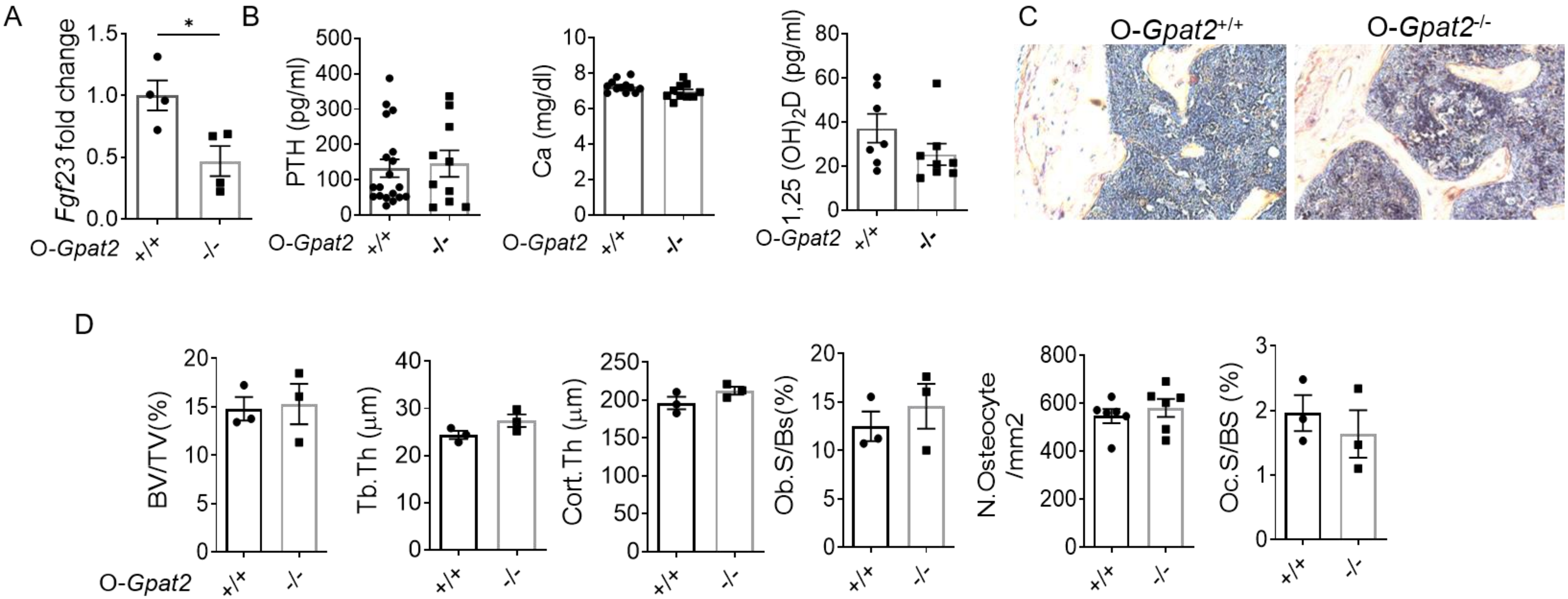

Supplemental Figure 2. Baseline bone mineral parameters in O-*Gpat2*$^{-/-}$ mice. (**A**) Bone *Fgf23* gene expression relative to GAPDH, as measured by qPCR in O-*Gpat2*$^{+/+}$ and O-*Gpat2*$^{-/-}$ mice. (**B**) Plasma parathyroid hormone (PTH), calcium (Ca), and 1,25-dihydroxyvitamin D_3 (1,25$(OH)_2D_3$) levels in O-*Gpat2*$^{+/+}$ and O-*Gpat2*$^{-/-}$ mice. *n* = 7–18 mice per group, male and female, 12 weeks of age. (**C**) TRAP staining and (**D**) histomorphometric analysis of the distal femur (10× magnification) of O-*Gpat2*$^{+/+}$ and O-*Gpat2*$^{-/-}$ mice. *n* = 3 per group, male mice, 12 weeks of age. Data are mean ± SEM. Statistical significance was assessed by Welch's t test. BV/TV, bone volume/trabecular volume; Tb.Th, trabecular thickness; Cort. Th, cortical thickness; N.Ob/B.Pm, number of osteoblasts/bone perimeter; N. Osteocyte, number of osteocytes, N.Oc/B.Pm, number of osteoclasts/bone perimeter. * $p<0.05$

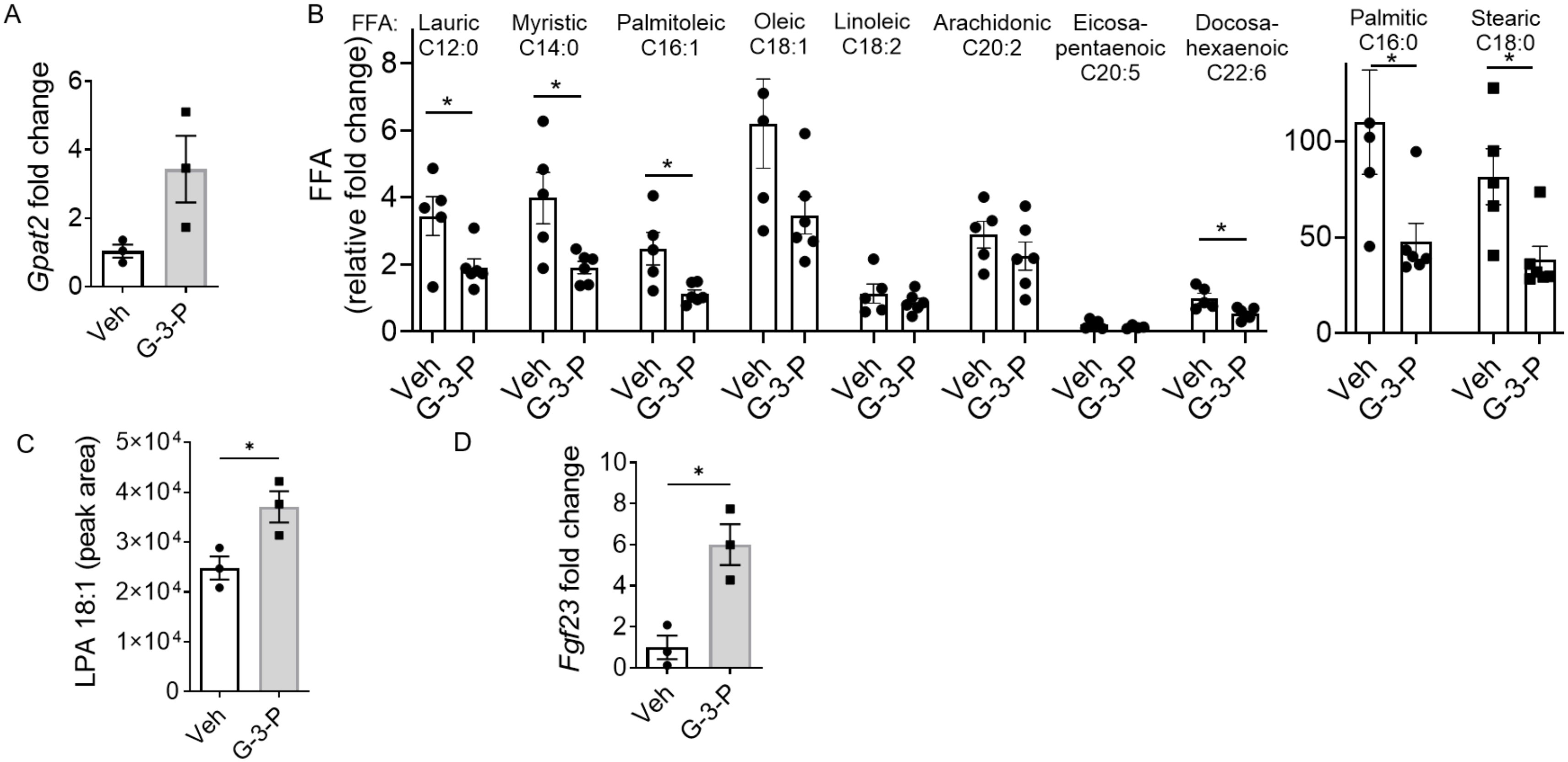

Supplemental Figure 3. Effect of glycerol-3-phosphate (G-3-P) on primary osteoblasts. (**A**) *Gpat2* mRNA expression, (**B**) intracellular free fatty acid levels, (**C**) lysophosphatidic acid 18:1 (LPA 18:1) levels, and (**D**) *Fgf23* gene expression in primary osteoblasts after 2 weeks of treatment with G-3-P (10 μM) compared to vehicle (Veh). n = 3-5 wells per group. Data are mean ± SEM. Statistical significance was assessed by Welch's t test. * $p<0.05$

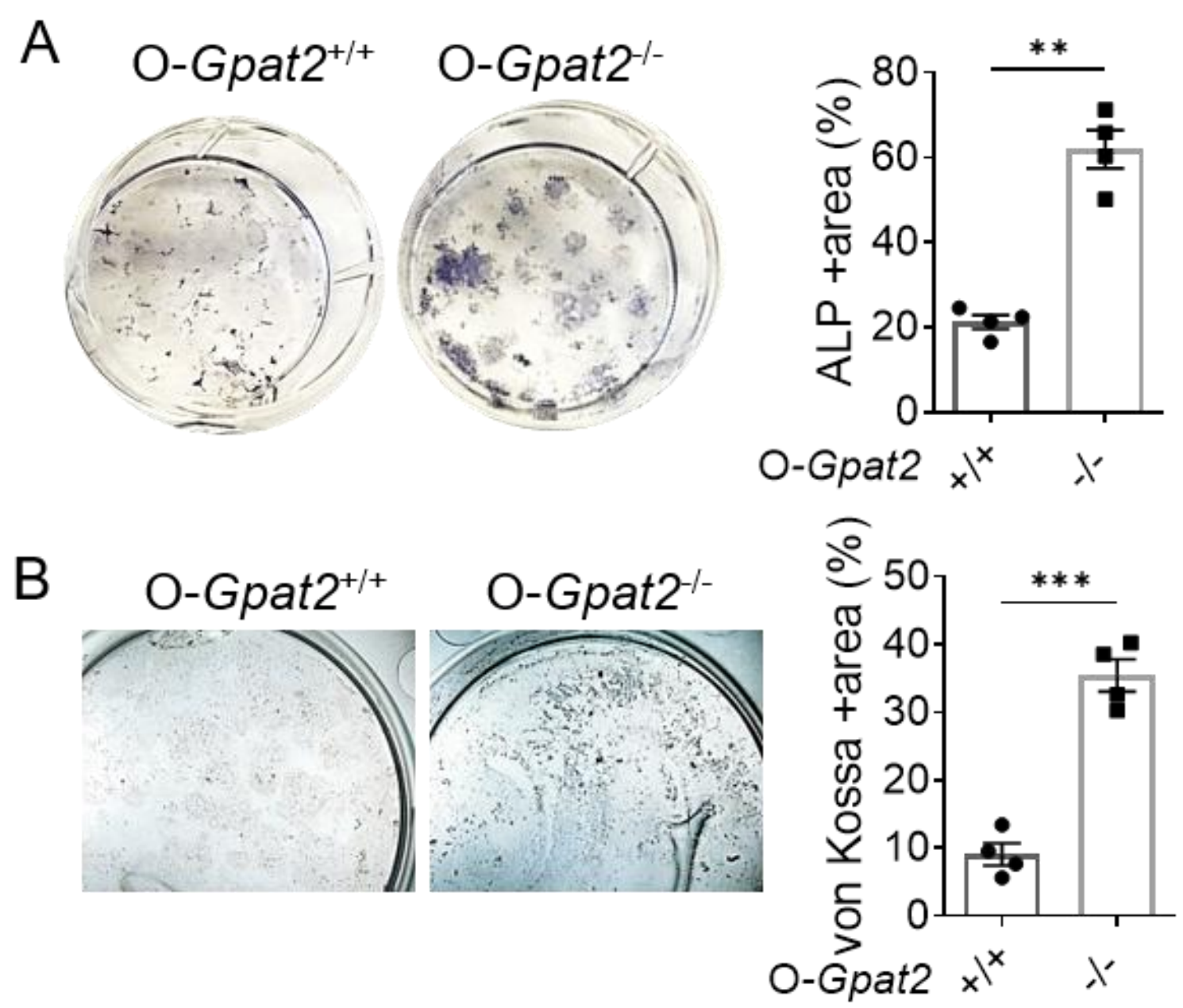

Supplemental Figure 4. Enhanced mineralization of O-*Gpat2*$^{-/-}$ primary osteoblasts. (**A**) Alkaline phosphatase staining with ImageJ quantification of positive area and (**B**) von Kossa staining with ImageJ quantification of mineralized area of primary osteoblasts from O-*Gpat2*$^{+/+}$ and O-*Gpat2*$^{-/-}$ mice. *n* = 4 wells per group. Data are mean ± SEM. Statistical significance was assessed by Welch's t test. $^{**}p<0.01$, $^{***}p<0.001$

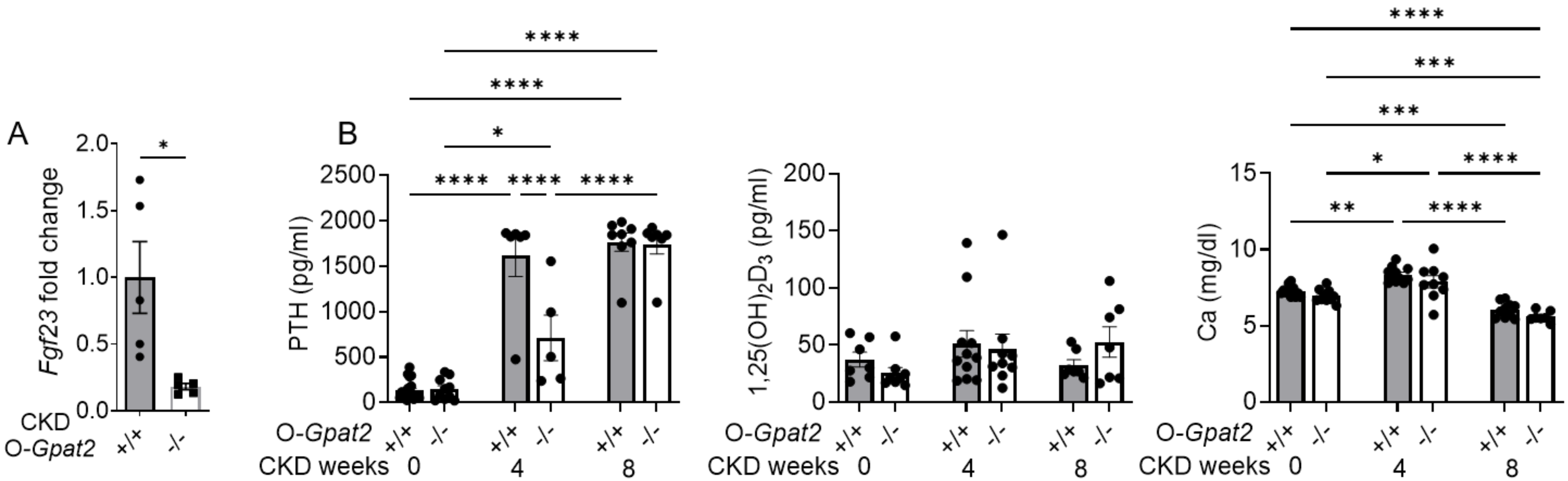

Supplemental Figure 5. Bone *Fgf23* expression and systemic parameters of mineral metabolism in mice with osteoblast specific *Gpat2* deletion during CKD. (**A**) qPCR *Fgf23* expression in the bone relative to GAPDH in O-*Gpat2*$^{+/+}$ and O-*Gpat2*$^{-/-}$ mice following 8 weeks of CKD. (**B**) Plasma parathyroid hormone (PTH), 1,25 dihydroxyvitamin D3 (1,25(OH)$_2$D3) and calcium (Ca) levels in O-*Gpat2*$^{+/+}$ and O-*Gpat2*$^{-/-}$ mice following 8 weeks of adenine diet–induced CKD. Data are mean ± SEM. Statistical significance was determined by Welch's t test (A) or two-way ANOVA (B). *n* = 5–12 mice per group, male and female, 12-20 weeks of age. * $p<0.05$, ** $p<0.01$, *** $p<0.001$, **** $p<0.0001$

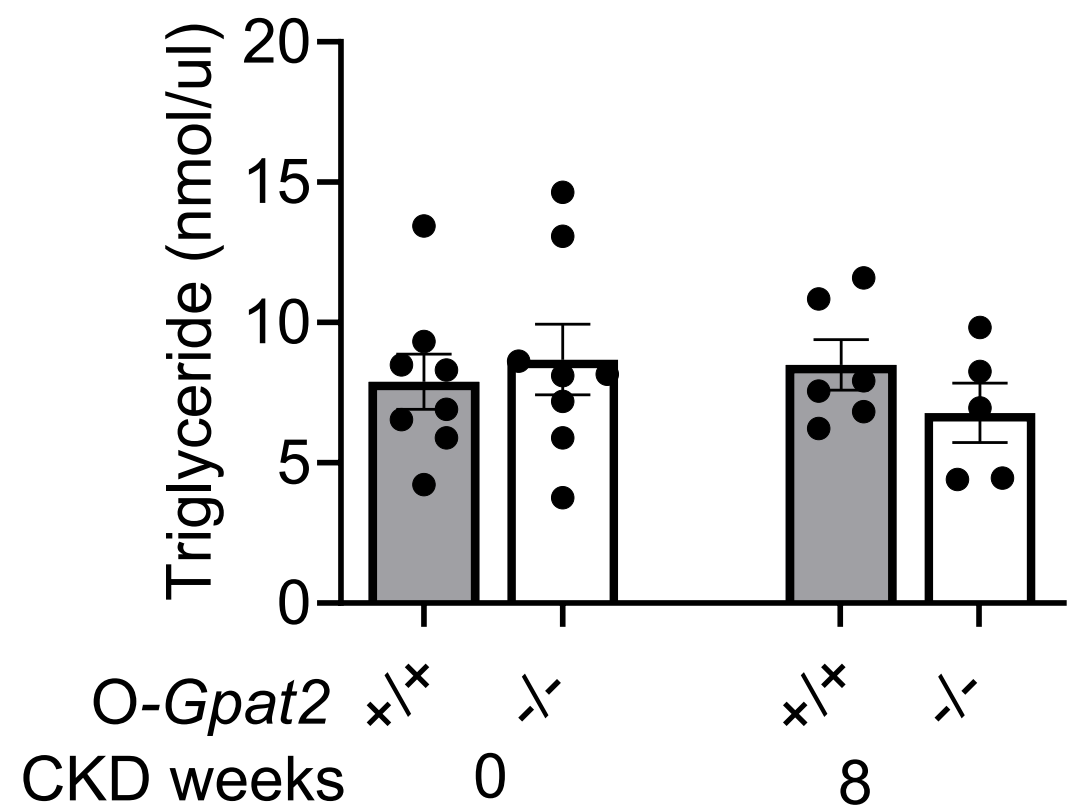

Supplemental Figure 6. Plasma triglycerides in O-*Gpat2*$^{+/+}$ and O-*Gpat2*$^{-/-}$ mice. Plasma triglyceride levels in O-*Gpat2*$^{+/+}$ and O-*Gpat2*$^{-/-}$ mice at baseline and after 8 weeks of adenine diet–induced CKD. Data are mean ± SEM. Statistical significance was determined by two-way ANOVA. *n* = 6 mice per group, male and female, 20 weeks of age.

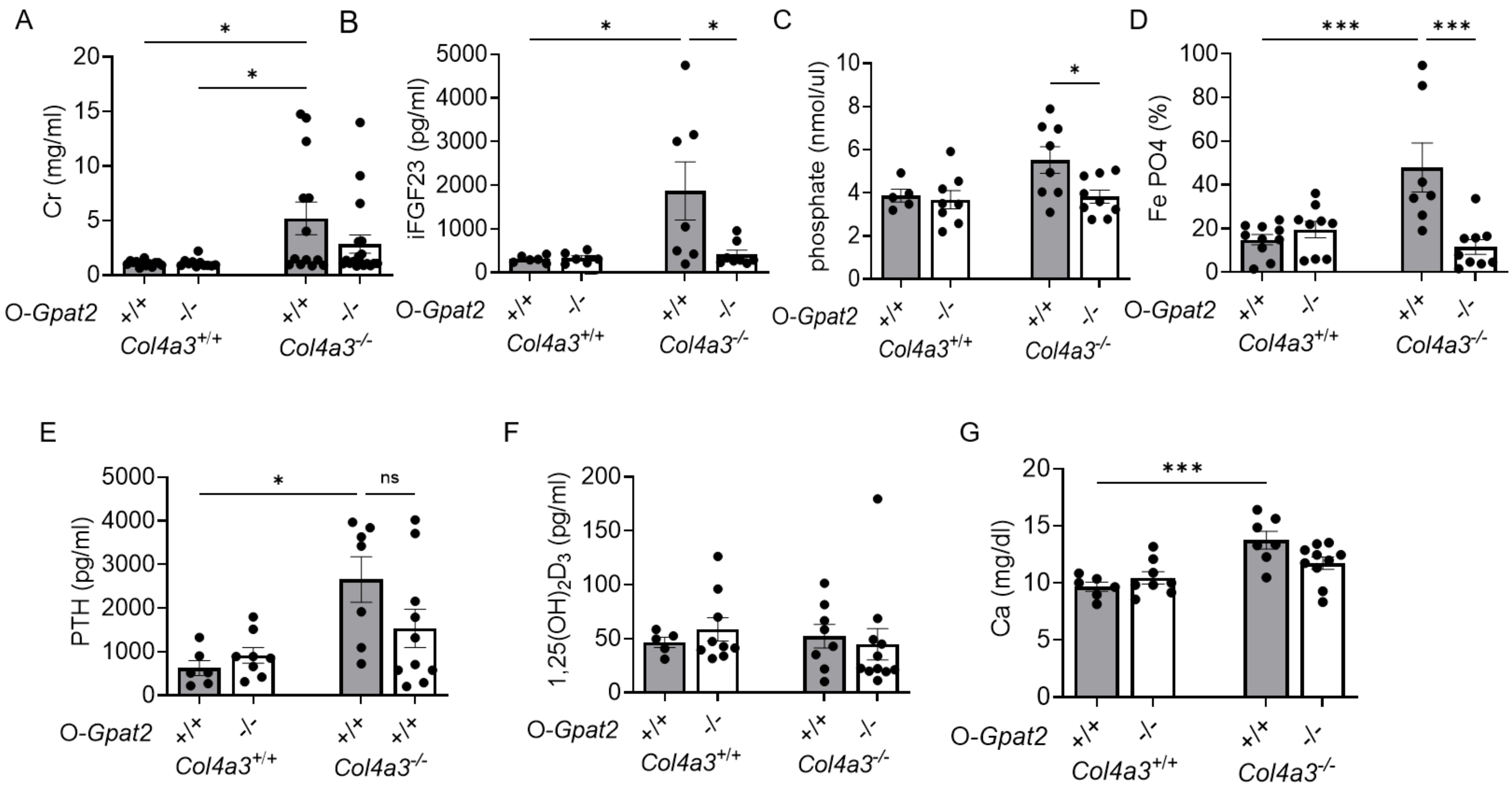

Supplemental Figure 7. Osteoblast-specific *Gpat2* deletion preserves systemic mineral metabolism in *Col4a3*$^{-/-}$ (Alport) mice. Plasma (**A**) creatinine, (**B**) intact FGF23 (iFGF23), (**C**) phosphate, (**D**) fractional excretion of phosphate ($FEPO_4$), (**E**) PTH, (**F**) 1,25 dihydroxyvitamin D3 (1,25$(OH)_2$D3), and (**G**) calcium (Ca) in O-*Gpat2*$^{+/+}$ and O-*Gpat2*$^{-/-}$ *Col4a3*$^{+/+}$ and O-*Gpat2*$^{+/+}$ and O-*Gpat2*$^{-/-}$ *Col4a3*$^{-/-}$ mice at 12 weeks of age. Data are mean ± SEM. Statistical significance was determined by two-way ANOVA. *n* = 6–8 per group, male mice. * *p*<0.05, ****p*<0.001

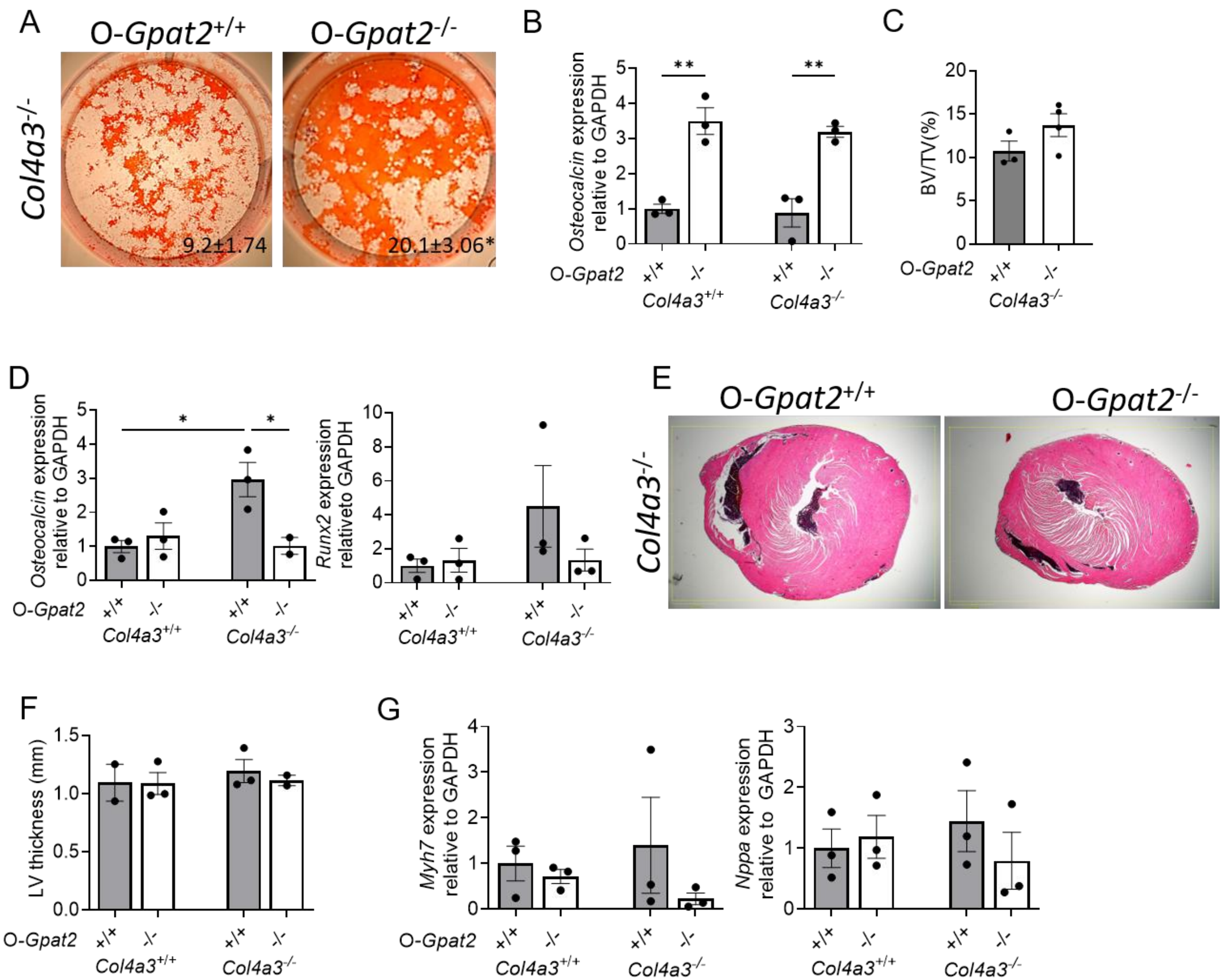

Supplemental Figure 8. Osteoblast *Gpat2* deletion improves bone mineralization and reduces vascular calcification in *Col4a3*$^{-/-}$ mice. (**A**) Alizarin Red staining and (**B**) *osteocalcin* qPCR gene expression of primary osteoblasts from O-*Gpat2*$^{+/+}$ and O-

Gpat2$^{-/-}$ *Col4a3*$^{-/-}$ mice at 12 weeks of age. (**C**) Bone volume / trabecular volume (BV/TV) of distal femurs, (**D**) vascular expression of osteogenic genes (*osteocalcin* and *Runx2*), (**E**) Hematoxylin and eosin staining of left ventricular cross-sections (10× magnification), (**F**) left ventricular (LV) wall thickness, and (**G**) qPCR analysis of cardiac hypertrophy genes (*Myh7* and *Nppa*) from O-*Gpat2*$^{+/+}$ and O-*Gpat2*$^{-/-}$ *Col4a3*$^{+/+}$ and O-*Gpat2*$^{+/+}$ and O-*Gpat2*$^{-/-}$ *Col4a3*$^{-/-}$ mice mice at 12 weeks of age. Data are mean ± SEM. Statistical significance was determined by two-way ANOVA, except (**C**) Welch's t test. *n* = 6–8 per group, male mice, 12 weeks of age. * $p<0.05$, **$p<0.01$

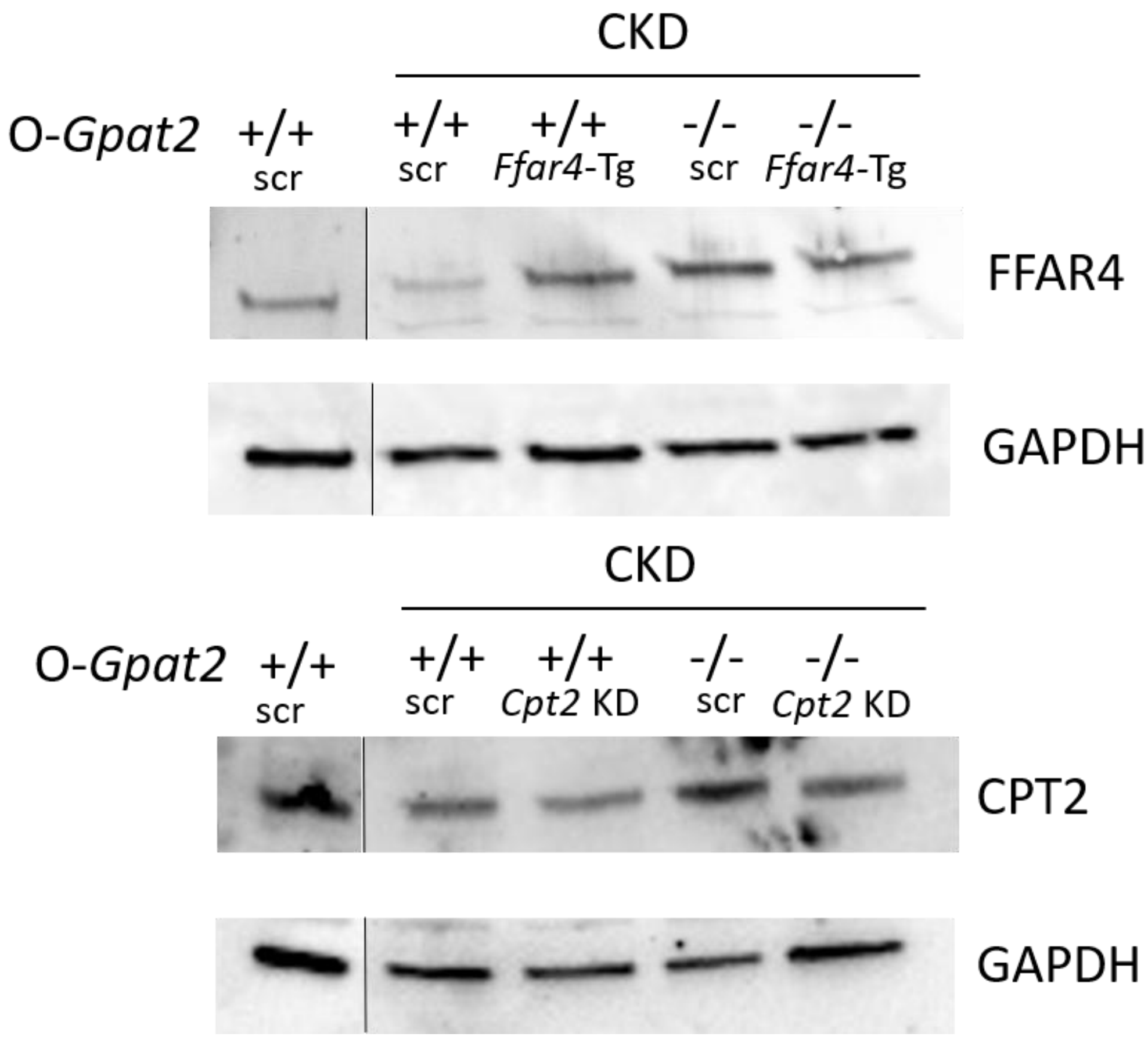

Supplemental Figure 9. Modulation of lipid metabolism proteins in CKD osteoblasts. Representative immunoblots for FFAR4, CPT2, and GAPDH in primary osteoblasts from control and adenine diet–induced CKD mice. Scr, scramble, *Ffar4*-Tg, *Ffar4* overexpression, *Cpt2* KD, *Cpt2* knock-down.

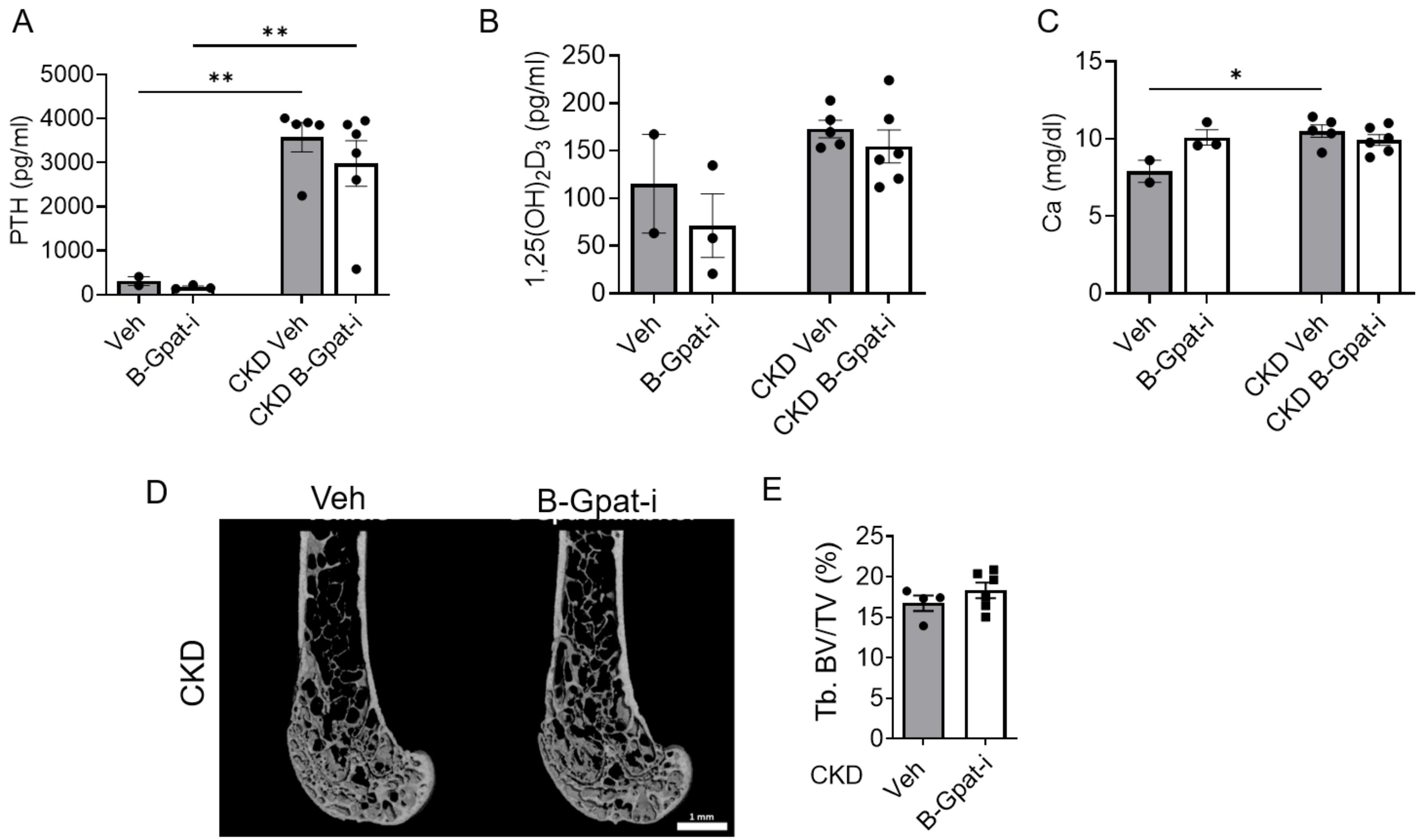

Supplemental Figure 10. Systemic mineral metabolism following bone-targeted GPAT inhibition. Plasma (**A**) PTH, (**B**) $1,25(OH)_2D_3$, and (**C**) calcium (Ca) levels and (**D**) representative μCT images of sagital sections of distal femur and (**E**) trabecular bone volume/trabecular volume (BV/TV) in mice treated with bone-targeted GPAT inhibitor (bGPATi) or vehicle after 4 weeks of adenine diet–induced CKD. Data are mean ± SEM. Statistical significance was determined by two-way ANOVA (**A**-**C**) or Welch's t test (**E**). *n* = 6–8 per group, male mice, 16 weeks of age. * $p<0.05$, ** $p<0.01$

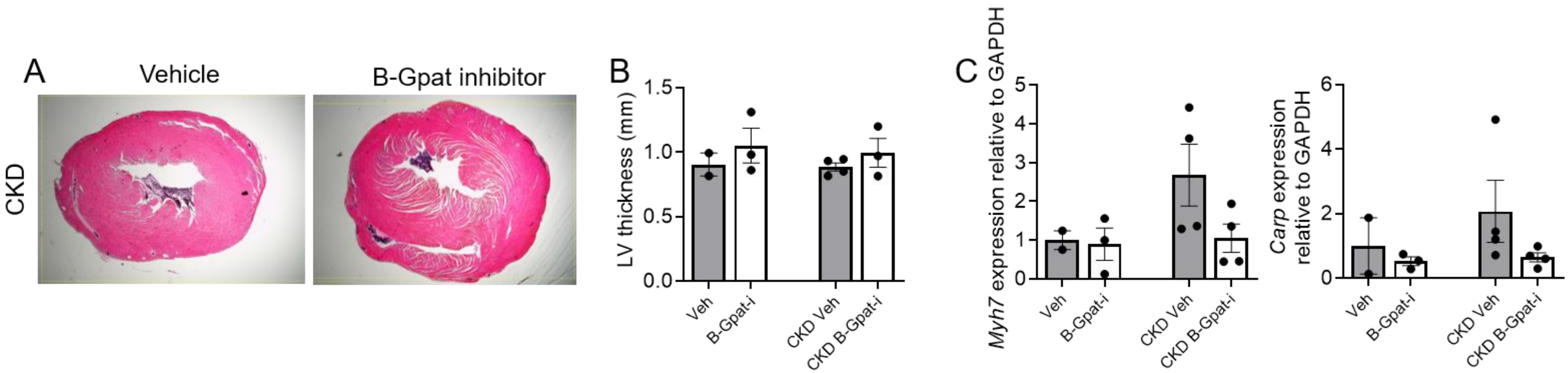

Supplemental Figure 11. Cardiac phenotype following bone-targeted GPAT inhibition in CKD. (**A**) Hematoxylin and eosin staining of left ventricular cross-sections (10× magnification), (**B**) left ventricular wall thickness, and (**C**) qPCR analysis of cardiac hypertrophy genes (*Myh7* and *Carp*) in mice treated with bone-targeted GPAT inhibitor (bGPATi) or vehicle after 4 weeks of adenine diet–induced CKD. Data are mean ± SEM. Statistical significance was determined by two-way ANOVA (**B**, **C**). *n* = 6–8 per group, male mice, 12 weeks of age.

Supplemental Table 1. Histomorphometry of O-*Gpat2*$^{+/+}$ and O-*Gpat2*$^{-/-}$ and control femurs at 8 weeks of CKD. BV/TV (bone volume/trabecular volume), Tb.N (trabecular number), Tb.Th (trabecular thickness, Tb.Sp (trabecular separation), N.Ob/B.Pm (number of osteoblasts/bone perimeter), Ob.S/Bs (osteoblast surface/bone surface), N.Oc/B.Pm (number of osteoclasts/bone perimeter), Oc.S/BS (osteoclast surface/bone surface), ES/BS (erosion surface/bone surface), N. Ad/T.Ar (number of adipocytes/ trabecular area), Ad.Ar/T.Ar (adipocyte area/trabecular area), MAR-Tb (mineral apposition rate – trabecular), MAR-Cort (mineral apposition rate – cortical), BFR/BS (bone formation rate/bone surface), O.Th-Tb (osteoid thickness – trabecular), O.Th_Cort (osteoid thickness – cortical). *P* values by Welch's *t*-test. Data average ± SD, *n* = 5 per group, male mice at 20 weeks of age.

	O-Gpat2$^{+/+}$	*O-Gpat2*$^{-/-}$	*P* value
**Bone:**			
BV/TV (%)	3.2±0.5	6.8±1.0	0.013
Tb.N (/mm)	1.0±0.1	1.9±0.3	0.018
Tb.Th (µm)	30.5±2.4	34.4±1.2	0.126
Tb.Sp (µm)	1003.5±143.5	535.2±83.7	0.010
**Osteoblasts:**			
N.Ob/BPm (/mm)	5.2±0.8	8.7±1.3	0.044
Ob.S/Bs (%)	8.0±1.6	14.5±3.0	0.088
Osteocytes:			
N osteocytes (/mm^2)	532±110	579±124	0.655
Osteoclasts:			
N.Oc/BPm (/mm)	1.3±0.2	1.3±0.2	0.984
Oc.S/BS (%)	2.7±0.4	2.8±0.2	0.859
ES/BS (%)	1.3±0.3	1.1±0.2	0.680
Adipocytes:			
N.Ad/T.Ar (/mm)	41.7±18.2	41.4±8.2	0.986
Ad.Ar/T.Ar (%)	3.0±1.6	2.5±0.5	0.762
**Mineral apposition:**			
MAR-Tb (µm/d)	0.66±0.04	0.76±0.04	0.030
MAR-Cort (µm/d)	0.82±0.04	1.00±0.04	0.004
BFR/BS (µm^3/µm^2/d)	0.6±0.02	0.8±0.06	0.046
**Osteoid:**			
O.Th-Tb (µm)	1.51±0.07	1.07±0.48	0.378
O.Th-Cort (µm)	1.62±0.19	1.02±0.12	0.034

Supplemental Table 2. Cardiac ultrasound parameters in O-*Gpat2*$^{+/+}$ and O-*Gpat2*$^{-/-}$ mice with CKD. IVSd, interventricular septal thickness in diastole; LVIDd, left ventricular internal diameter in diastole; LVPWd, left ventricular posterior wall thickness in diastole; EF, ejection fraction. Values are presented as average ± SD. *p<0.05 vs CKD O-*Gpat2*$^{+/+}$; #p<0.05 vs non-CKD O-*Gpat2*$^{+/+}$, two-way ANOVA, *n* = 4 mice per group (male) after 12 weeks of adenine diet-induced CKD

	IVSd (mm)	LVIDd (mm)	LVPWd (mm)	EF (%)
O-Gpat2$^{+/+}$	0.85±0.06	3.15±0.30	0.90±0.33	83.81±3.75
O-Gpat2$^{-/-}$	0.83±0.06	3.27±0.03	1.11±0.19	79.33±4.81
CKD *O-Gpat2*$^{+/+}$	1.20±0.16$^{\#}$	2.25±0.37$^{\#}$	1.33±0.22	84.89±6.56
CKD *O-Gpat2*$^{-/-}$	0.83±0.12*	2.77±0.62	1.08±0.21	88.53±7.70

Supplemental Table 3. Primer sequences used for qPCR and PCR analyses

**qPCR Primer**	**Forward Primer**	**Reverse Primer**
Gpat2	CTCTTGTGGCCCTTTTCCAG	TGTCCCCTTGTGTGGAGTTG
Osteocalcin (bglap)	CTGACCTCACAGATCCCAAGC	TGGTCTGATAGCTCGTCACAAG
Runx2	ATGCTTCATTCGCCTCACAAA	GCACTCACTGACTCGGTTGG
Alp	ACCTTCATCGGAAACTCCAAAG	ACTGTTAGGCTCAGGTGAACT
Osx (Sp7)	ATGGCGTCCTCTCTGCTTG	TGAAAGGTCAGCGTATGGCTT
Myh7	ACTGTCAACACTAAGAGGGTCA	TTGGATGATTTGATCTTCCAGGG
Carp	GCTGGTAACAGGCAAAAAGAAC	CCTCTCGCAGTTTCTCGCT
Nppa	GCTTCCAGGCCATATTGGAG	GGGGGCATGACCTCATCTT
Fgf23	ATGCTAGGGACCTGCCTTAGA	GGAGCCAAGCAATGGGGAA
Gpat1	CTTGGCCGATGTAAACACACC	CTTCCGGCTCATAAGGCTCTC
Gpat2	AGCAGAGGAGTAACCACAATG	GGGCGATACTTTCCCAGGA
Gpat3	CGGATTATCCCTGGGTATCTCG	CGAAGTCCCTTCCTCGAAGAC
Gpat4	CCGTTGGTGTAGGGCTTGT	AACCTCCTGGGTATCTCCCTG
Gapdh	TAACATCAAATGGGGTGAGG	GGTTCACACCCATCACAAAC
**PCR Primer**	**Forward Primer**	**Reverse Primer**
Gpat2	CTCTTGTGGCCCTTTTCCAG	TGTCCCCTTGTGTGGAGTTG
Cre	CGCGGTCTGGCAGTAAAAACTATC	CCCACCGTCAGTACGTGAGATATC
