# Supplementary material for: Targeting osteoblast fatty acid metabolism attenuates skeletal and cardiovascular complications in chronic kidney disease models
Source: J Clin Invest. 2026 Sep 29;136(22):e207322. doi: 10.1172/JCI207322 (PMC13767616; doi:10.1172/JCI207322)
Supplement: Supplementary file 2 [file jci-136-207322-s154.pdf]

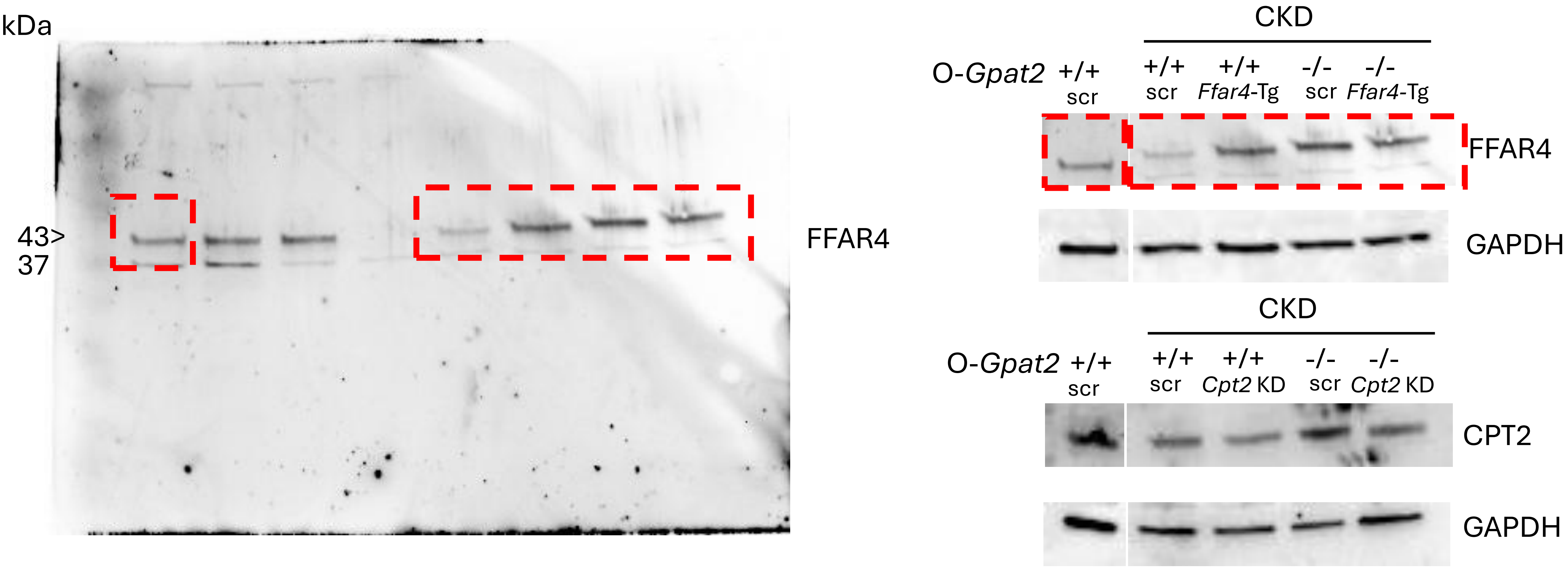

Full unedited gel for Supplementary Fig 9.

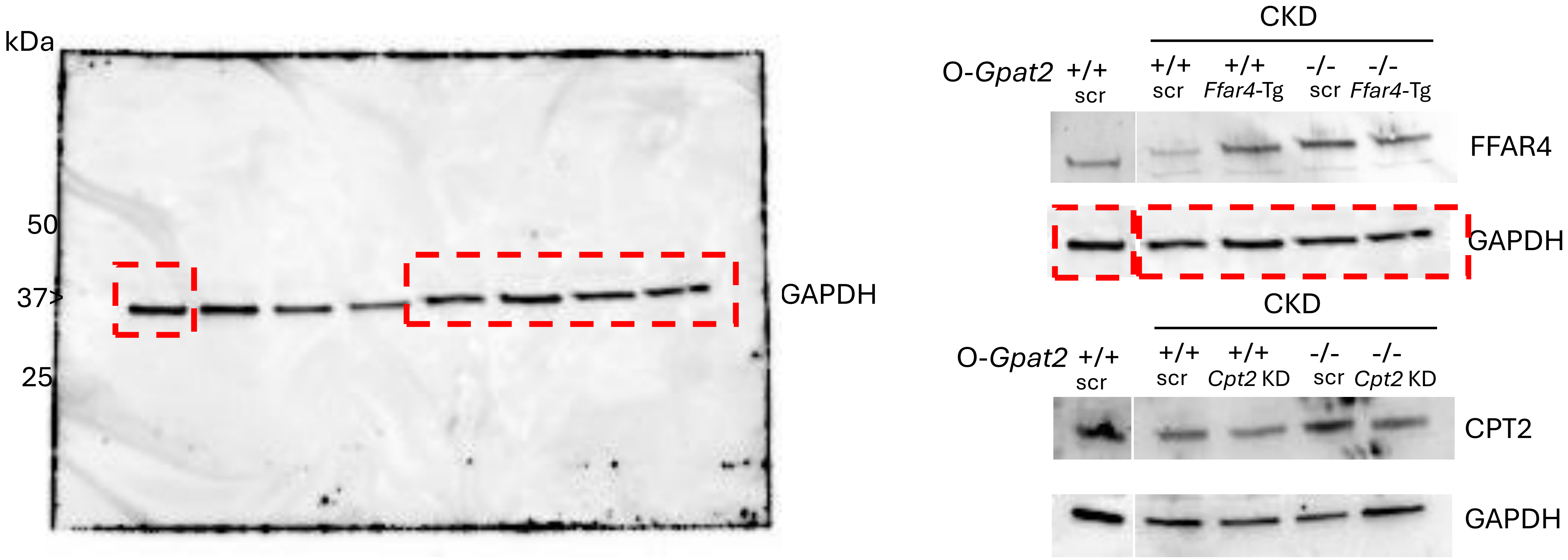

Full unedited gel for Supplementary Fig 9.

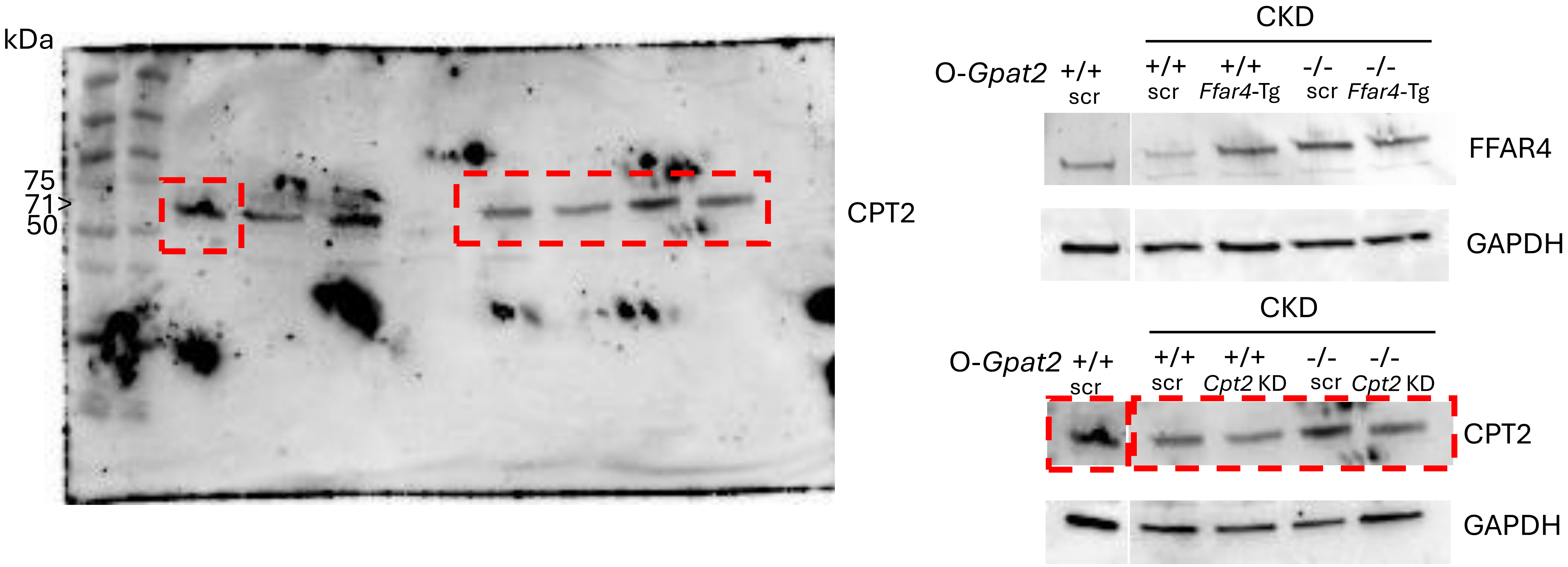

Full unedited gel for Supplementary Fig 9.

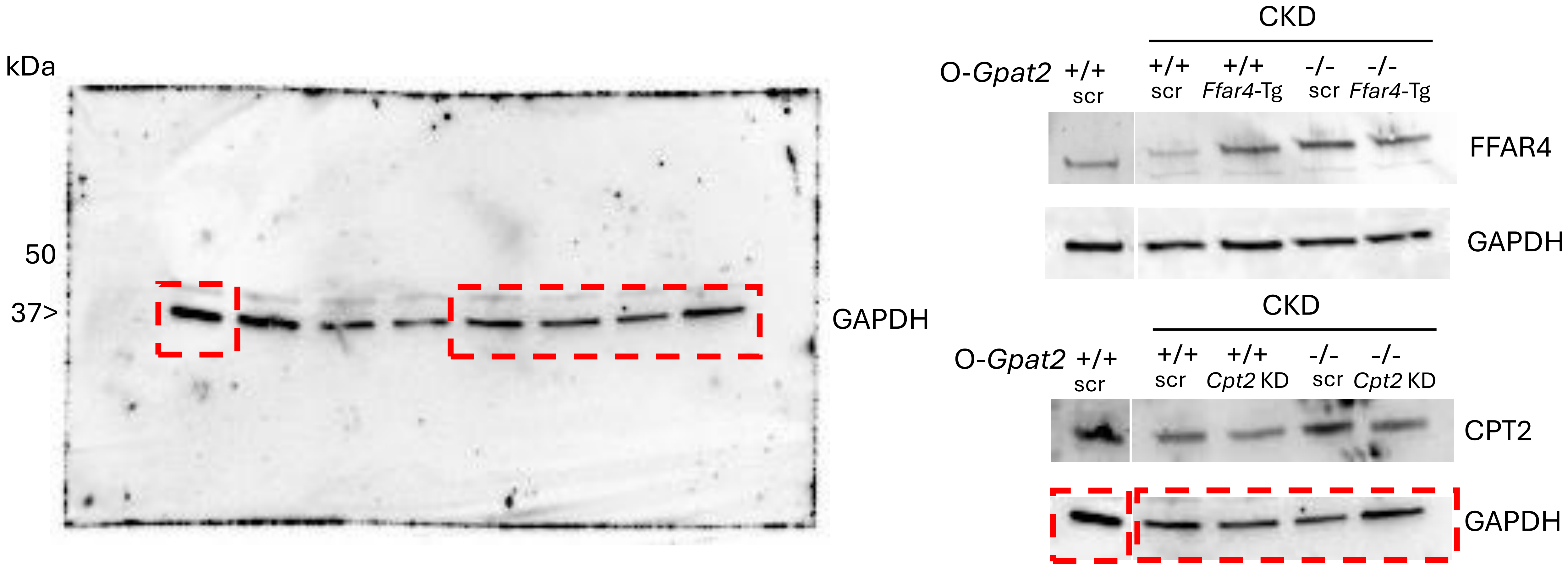

Full unedited gel for Supplementary Fig 9.
